# *Populus cathayana* genome and population resequencing provide insights into its evolution and adaptation

**DOI:** 10.1093/hr/uhad255

**Published:** 2023-12-11

**Authors:** Xiaodong Xiang, Xinglu Zhou, Hailing Zi, Hantian Wei, Demei Cao, Yahong Zhang, Lei Zhang, Jianjun Hu

**Affiliations:** State Key Laboratory of Tree Genetics and Breeding, Key Laboratory of Tree Breeding and Cultivation of National Forestry and Grassland Administration, Research Institute of Forestry, Chinese Academy of Forestry, Beijing 100091, China; Co-Innovation Center for Sustainable Forestry in Southern China, Nanjing Forestry University, Nanjing, Jiangsu 210037, China; State Key Laboratory of Tree Genetics and Breeding, Key Laboratory of Tree Breeding and Cultivation of National Forestry and Grassland Administration, Research Institute of Forestry, Chinese Academy of Forestry, Beijing 100091, China; Co-Innovation Center for Sustainable Forestry in Southern China, Nanjing Forestry University, Nanjing, Jiangsu 210037, China; Novogene Bioinformatics Institute, Beijing 100083, China; State Key Laboratory of Tree Genetics and Breeding, Key Laboratory of Tree Breeding and Cultivation of National Forestry and Grassland Administration, Research Institute of Forestry, Chinese Academy of Forestry, Beijing 100091, China; State Key Laboratory of Tree Genetics and Breeding, Key Laboratory of Tree Breeding and Cultivation of National Forestry and Grassland Administration, Research Institute of Forestry, Chinese Academy of Forestry, Beijing 100091, China; State Key Laboratory of Tree Genetics and Breeding, Key Laboratory of Tree Breeding and Cultivation of National Forestry and Grassland Administration, Research Institute of Forestry, Chinese Academy of Forestry, Beijing 100091, China; State Key Laboratory of Tree Genetics and Breeding, Key Laboratory of Tree Breeding and Cultivation of National Forestry and Grassland Administration, Research Institute of Forestry, Chinese Academy of Forestry, Beijing 100091, China; Co-Innovation Center for Sustainable Forestry in Southern China, Nanjing Forestry University, Nanjing, Jiangsu 210037, China; State Key Laboratory of Tree Genetics and Breeding, Key Laboratory of Tree Breeding and Cultivation of National Forestry and Grassland Administration, Research Institute of Forestry, Chinese Academy of Forestry, Beijing 100091, China; Co-Innovation Center for Sustainable Forestry in Southern China, Nanjing Forestry University, Nanjing, Jiangsu 210037, China

## Abstract

*Populus cathayana* Rehder, an indigenous poplar species of ecological and economic importance, is widely distributed in a high-elevation range from southwest to northeast China. Further development of this species as a sustainable poplar resource has been hindered by a lack of genome information the at the population level. Here, we produced a chromosome-level genome assembly of *P. cathayana*, covering 406.55 Mb (scaffold N50 = 20.86 Mb) and consisting of 19 chromosomes, with 35 977 protein-coding genes. Subsequently, we made a genomic variation atlas of 438 wild individuals covering 36 representative geographic areas of *P. cathayana*, which were divided into four geographic groups. It was inferred that the Northwest China regions served as the genetic diversity centers and a population bottleneck happened during the history of *P. cathayana*. By genotype–environment association analysis, 947 environment-association loci were significantly associated with temperature, solar radiation, precipitation, and altitude variables. We identified local adaptation genes involved in DNA repair and UV radiation response, among which *UVR8*, *HY5*, and *CUL4* had key roles in high-altitude adaptation of *P. cathayana*. Predictions of adaptive potential under future climate conditions showed that *P. cathayana* populations in areas with drastic climate change were anticipated to have greater maladaptation risk. These results provide comprehensive insights for understanding wild poplar evolution and optimizing adaptive potential in molecular breeding.

## Introduction

The genus *Populus* (Salicaceae) is widely distributed in the northern hemisphere throughout the subtropical to boreal forests, and is classified into six sections (*Abaso*, *Aigeiros*, *Leucoides*, *Populus*, *Tacamahaca*, and *Turanga*) [1]. China is a unique region concerning the genus *Populus*, with about half of *Tacamahaca* poplars having their natural range completely or partlyin China [2]. *Populus cathayana* Rehder (Salicaceae, *Tacamahaca*) is a native tree in China, widely distributed in northern, central, and southwestern areas [3, 4]. This species predominantly grows at altitudes of 1000–3000 m, with some occurring at 3900 m on the eastern margin of the Qinghai–Tibet Plateau [5]. Despite the challenging climates of high altitudes and short growing seasons, *P. cathayana* demonstrates exceptional growth potential and high adaptability [6]. Wild *P. cathayana* is commonly used as a hybrid parent to culture elite poplar varieties that adapt to harsh environments and have beneficial traits such as fast growth and cold tolerance.

Disentangling the genetic mechanisms driving local adaptation and genetic differentiation is essential for conservation efforts and the development of breeding strategies [7, 8]. Extensive experimental research on tree populations has shown that local populations consistently exhibit higher adaptability in their native habitats [9, 10]. Local adaptation related to spatial variations in natural selection pressures can be explained by factors such as latitude, longitude, altitude, and slope [11, 12]. The distribution of populations is significantly influenced by dramatic climatic oscillations and historical geology, leaving diagnostic signatures in the genomes [13]. The use of population genetics methods and genomic data can be a crucial strategy to examine local adaptation across wild species [14]. High-quality genomes of poplars such as *Populus trichocarpa, P. alba*, *P. euphratica*, and *P. alba* × *P. glandulosa* provide a foundation for understanding adaptive evolution and genetic differentiation [15–18]. Wild populations of *P. cathayana* were considered to be important genetic resources in meeting ecological and forest product needs [4]. However, previous studies have used limited markers, and have not provided understanding of the genomic genetic variation of *P. cathayana*.

Future global environmental changes are predicted to negatively affect ecosystem homeostasis, and climate change increases our interest in the adaptation of species and populations to new environments [12]. Trees are long-lived organisms with slow adaptive evolution, making them vulnerable to rapid environmental changes. In recent years, researchers have increasingly used genomic methods to predict the impact of climate change on local adaptation and species vulnerability [19]. Genotype–environmental association (GEA) is used as an approach to elucidate the genetic bases of adaptation of *Betula nana* [20], *Thlaspi arvense* [21], *Quercus lobata* [22], *Picea sitchensis* [23], and *Populus trichocarpa* [24]. Due to the polygenic nature of these adaptive variants, the genetic architecture of local adaptation to climate can be very diverse among even closely related species. For the identified key adaptive mutation sites, genomic shifts can be measured to assess the amount of population genetic composition change required to track future environmental conditions [25, 26]. Therefore, the use of genomic tools can not only simulate changes in species ranges over time but also provide novel understanding to assess evolutionary adaptation potential and the ability to withstand future climate risks.

*Populus cathayana* has been recognized for its high-altitude growth and wide adaptation to harsh environmental conditions, such as low temperatures and intense solar radiation. However, the genetic mechanisms of local adaptation of *P. cathayana* at broad spatial scales are still unknown. As a wild tree species population, it has rarely been subjected to artificial selection, making it an ideal model for understanding species adaptability and local adaptation. Here, we assembled a *de novo* chromosome-scale *P. cathayana* genome and resequenced the genomes of 438 individuals from 36 geographic regions in China. The population structure, genetic diversity, and demographic processes of the *P. cathayana* population were revealed by genome-wide variants and phylogenetic analysis. Based on these genomic datasets, we uncovered genetic evidence of the environmental adaptability of *P. cathayana*. This study offers new insights into genome evolution and adaptation in a major forest species, and serve as a valuable resource for genome-based poplar improvement.

## Results

### *Populus cathayana* genome assembly and annotation

The genome of *P. cathayana* was sequenced, and 60.71 Gb of PacBio long-read sequencing data (~149×), 47.35 Gb Illumina data (~116×), and 50.63 Gb of Hi-C (chromosome conformation capture) paired-end reads data (~124×) were obtained for *de novo* genome assembly (Supplementary Data Tables S1 and S2). The estimated size of the nuclear genome of *P. cathayana* by flow cytometry and *K*-mer analysis was ~412.4 and ~423.7 Mb, respectively (Supplementary Data Fig. S1, Supplementary Data Table S3). Based on PacBio and Illumina data, we obtained an initial genome assembly of 411 Mb (Supplementary Data Table S4). The scaffold extension and chromosomal mapping were performed using Hi-C data, resulting in a final assembly of the *P. cathayana* genome with a size of 406.5 Mb (Table 1). This assembly had 21 scaffolds, with a scaffold N50 of 20.86 Mb (Table 1, Supplementary Data Table S5). Specifically, 405.9 Mb (99.84%) of the assembly was successfully ordered and oriented to 19 pseudo-chromosomes (Supplementary Data Fig. S2, Supplementary Data Table S6). The 97.96% Benchmarking Universal Single-Copy Orthologs (BUSCO) and 98.25% Core Eukaryotic Genes Mapping Approach (CEGMA) could be completely detected in the assembly. In addition, 95.99% of Illumina reads were also mapped to the assembled genome, which indicated that the genome assembly was high-quality (Supplementary Data Tables S7 and S8).

**Table 1:** Statistics for genome assembly and annotation for *P. cathayana*.

**Assembly feature**	**Statistic**
Assembly	
Genome size (bp)	406 549 807
Number of scaffolds	21
N50 of scaffolds (bp)	20 860 933
Chromosome-scale scaffolds (bp)	405 899 219 (99.84%)
Number of contigs	77
N50 of contigs (bp)	10 281 541
Number of gaps	56
Complete BUSCOs	1581 (97.96%)
GC content of genome (%)	33.84
Annotation	
Number of predicted protein-coding genes	35 977
Average gene length (bp)	3444
Average coding sequence length (bp)	1317
Average exons per transcript	5.4
Repeat sequences (bp)	173 824 474 (42.76%)

**Figure 1:**
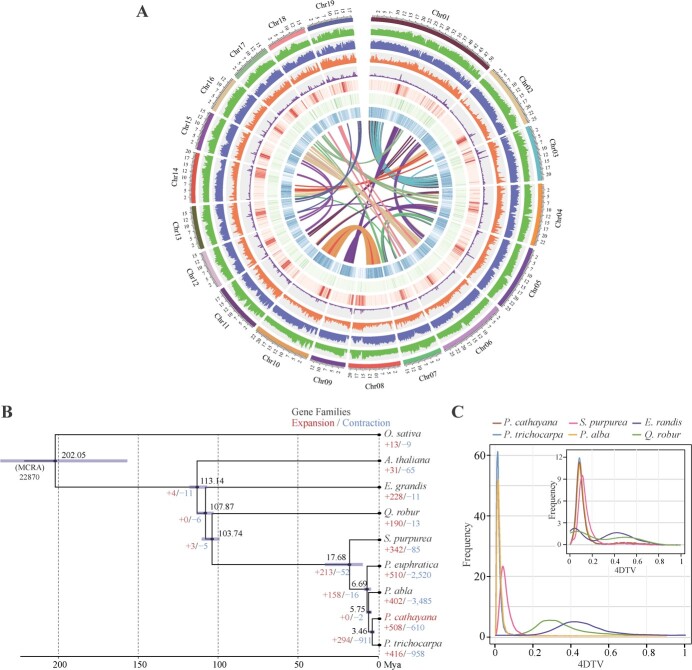
Evolutionary and collinearity analyses of the *P. cathayana* genome. **A** Tracks from outside to inside represent chromosomes, density of SNPs, indels, CNVs, SVs, Gypsy LTR-RT density, Copia LTR-RT density, gene density, and genomic collinearity. Variant density was calculated in non-overlapping 100-kb windows. **B** Phylogenetic tree, divergence time, and expansion and contraction of gene families for nine species. Evolutionary relationships and divergence times were calculated based on 805 single-copy orthologous genes. **C** The 4DTV distribution in the upper right (insert) shows 4DTV distribution from paralogs within *P. cathayana*, *P. trichocarpa*, *P. alba*, *Eucalyptus grandis*, *Salix purpurea*, and *Quercus robur*. The 4DTV distribution on the lower left shows the 4DTV distribution from orthologs between *P. cathayana* and the other five species.

In total, 35 977 protein-coding genes were predicted in the *P. cathayana* genome, with an average length of 3444 bp per gene and an average of 5.4 exons per transcript (Table 1, Supplementary Data Table S9). In addition, 35 366 (98.3%) genes could be annotated using functional databases (Supplementary Data Table S10). A total of 173.85 Mb sequences in the *P. cathayana* genome were annotated as repetitive sequences, comprising 150.9 Mb (37.11%) transposable repeats and 22.95 Mb (5.64%) tandem repeats (Supplementary Data Tables S11 and S12). Long-terminal repeats (LTRs) constituted the highest proportion of retrotransposons in the genome, accounting for 22.15%, with 9.69% of Gypsy superfamilies and 4.17% of Copia superfamilies dominating (Fig. 1A). In addition, a set of non-coding RNAs (rRNA, tRNA, miRNA, snRNA, and snoRNA) was identified (Supplementary Data Table S13).

### Genome evolution

**Figure 2:**
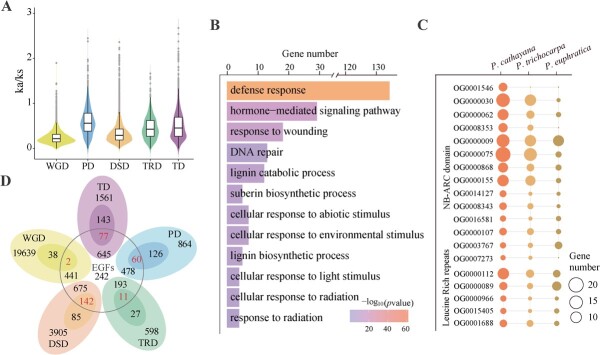
Genome duplication and evolution. **A***K*_a_/*K*_s_ ratio distributions of duplication gene pairs derived from five modes (WGD, TD, PD, TRD, and DSD). **B** Functional enrichment analysis of gene family expansion with *P. cathayana.***C** Numbers of NBS and NR-ARC domains in the *P. trichocarpa*, *P. euphratica*, and *P. cathayana* genomes. **D** Venn diagram showing overlap between members of EGFs and five duplicated types.

Utilizing 805 single-copy orthologs shared by nine species, we performed phylogenetic reconstruction and estimated species divergence time (Supplementary Data Table S14). *Populus cathayana* shared a common ancestor with four Salicaceae species, and the estimated divergence time was between 3.46 and 17.68 million years ago (Mya) (Fig. 1B). In four-fold synonymous third-codon transversion (4DTV) analysis, the appearance of sharp peaks of 4DTV in *P. cathayana*, *P. alba*, *P. trichocarpa*, and *Salix purpurea* represents an outbreak of gene duplication, corresponding to recent salicoid duplication (~65 Mya) and core eudicot triplication (~117 Mya). The distribution of 4DTV from orthologs indicated that divergence between *P. cathayana* and *P. trichocarpa* occurred ~4.1 Mya (4DTV ~0.01), which was consistent with the phylogenetic result (Fig. 1C, Supplementary Data Fig. S3). The 29 710 duplicated genes discovered in *P. cathayana* were categorized into five types: 20 120 whole-genome duplicates (WGDs, 67.7%), 2426 tandem duplicates (TDs, 8.2%), 1528 proximal duplicates (PDs, 5.1%), 829 transposed duplicates (TRDs, 2.8%), and 4807 dispersed duplicates (DSDs, 16.2%). The higher *K*_a_/*K*_s_ ratios were found in TD and PD types (Fig 2A), indicated that TDs and PDs underwent more rapid sequence divergence than genes originated from other duplication types.

All of the predicted gene models for the nine species were clustered into 29 525 orthogroups (Supplementary Data Fig. S4), of which 508 expanded and 610 contracted in *P. cathayana*. The expanded genes were significantly enriched in defense response, hormone signaling pathway, response to abiotic stimulus, and DNA repair (Fig. 2B, Supplementary Data Table S15). Specifically, the *P. cathayana* genomes exhibited an increase in genes related to defense, such as plant disease resistance (R) genes, containing NB-ARC and NBS domains (Fig. 2C). Among the members of expanded gene families (EGFs), 1260 (57.6%) genes originated as TD duplications and PD duplications, accounting for the largest proportion (Fig. 2D). The TD-EGF and PD-EGF gene-enriched categories were implicated in defense, response to stimulus, and stress (Supplementary Data Fig. S5). TDs and PDs have been identified as significant factors contributing to the expansion of gene families in *P. cathayana*, particularly in the case of newly formed duplications. These results demonstrated that gene family expansion plays a crucial role in the local adaption of *P. cathayana* during long-term evolution.

### Population structure and genetic diversity of *P. cathayana*

**Figure 3:**
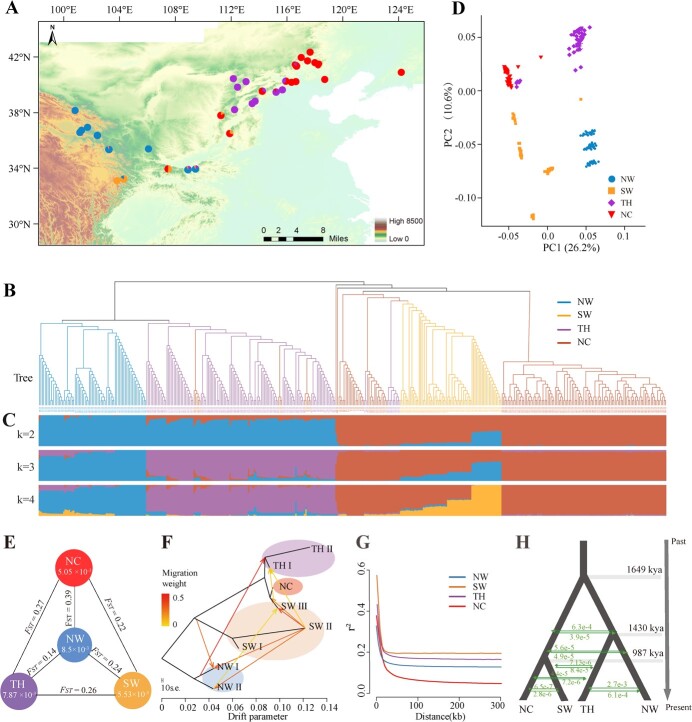
Geographic distribution, population structure, and genetic diversity of *P. cathayana* species. **A** Geographic distribution of sampling locations. The genetic composition at each sampling location is displayed as a pie chart, and the colors of the pies represent ancestral components according to the structure at *K* = 4. **B** Phylogenetic tree of all *P. cathayana* individuals. **C** Population structure of *P. cathayana* at cluster values (*K*) of 2–4. Each individual is denoted by a vertical bar composed of different colors corresponding to its proportion of genetic ancestry. More clusters values (*K* = 5–8) are shown in Supplementary Data Fig. S6. **D** PCA plot of the first two eigenvectors of the *P. cathayana* population. PC1 and PC2 divide the *P. cathayana* populations into four groups. **E** Nucleotide diversity (π) and population differentiation coefficient (*F*_ST_) among the four groups. **F** Population gene flow shown by TreeMix. Arrows indicate inferred gene flow events between populations, with direction from the source population to the recipient population. Gene flow is colored according to their weight. The classification of sample groups is shown in Supplementary Data Fig. S6. **G** LD decay for the four groups of *P. cathayana*. X-axis: physical distances between two SNPs; Y-axis: *R*^2^ used to measure linkage disequilibrium. **H** The best-fitting demographic model deduced by fastsimcoal2 is shown. Arrows represent estimated gene flow among clades.

We collected 438 individuals from 36 natural distribution regions of *P. cathayana* and generated whole-genome resequencing data (Fig. 3A, Supplementary Data Table S16). The cleaned reads were aligned to the *P. cathayana* genome, generating an average mapping rate of 93.36% and an average depth of 32.3 (Supplementary Data Table S17). We obtained 30 829 763 raw single-nucleotide polymorphisms (SNPs) and 12 374 210 high-quality SNPs after further filtering. A set of 569 435 insertion–deletions (indels), 35 416 copy-number variations (CNVs), and 9771 structure variants (SVs) was identified after filtering (Supplementary Data Table S18). Nearly half of the SNP variations were located within transposable elements (TEs), and the density of variations in TEs was higher than in mRNA regions (Supplementary Data Table S19).

The *P. cathayana* population could be divided into four groups based on the phylogenetic and population structure analyses, including NW (Northwest China), SW (Southwest China), TH (Tai-hang Mountains), and NC (North China) (Fig. 3B and C**,**Supplementary Data Fig. S6A and B). The four genetic groups that reflect the geographic distribution pattern were further supported by principal component analysis (PCA) (Fig. 3D). The Mantel test also revealed the strong significant correlation between geographic distance and genetic distance (Supplementary Data Fig. S6C). Moreover, we discovered population differentiation coefficient (*F*_ST_) values between the four groups that were high or moderate (0.14–0.39), which was consistent with population admixture (Fig. 3E). In addition, numerous subclades among closely related subgroups shared historical gene flow and ancestral variation (Fig. 3F). A clear signature of genetic admixture was present in the SW group, while extensive gene flow was detected between the SW and NW subclades. Allelic admixture in some areas was evident, probably due to the pollen transmission and reproduction history.

Among the *P. cathayana* genetic groups, the highest nucleotide diversity (π) was found in the NW group (π = 8.50 × 10^−3^), followed by the TH group (π = 7.87 × 10^−3^), NC group (π = 5.05 × 10^−3^), and SW group (π = 5.53 × 10^−3^) (Fig. 3E**,**Supplementary Data Table S17). Overall, the genetic diversity of the *P. cathayana* natural population was 6.1 × 10^−3^. The linkage disequilibrium (LD) of the whole population was 9.8 kb (*r*^2^ = 0.2); the NW group presented the fastest LD decay (9 kb), followed by the NC (11 kb) and TH (15 kb) groups (Fig. 3G). The SW group exhibited the slowest LD decay (22 kb) and had low nucleotide diversity. The *P. cathayana* population differentiation originated from the ancestor population [1649 thousand years ago (kya)], with TH and NW diverging from the common ancestor at ~1430 kya and SW and NC diverging at ~987 kya, as indicated by the demographic fluctuations model (Fig. 3H). The patterns of genomic diversity and population history strongly indicated an origin for *P. cathayana* in NW China.

### Demographic history and potential distribution of *P. cathayana*

**Figure 4:**
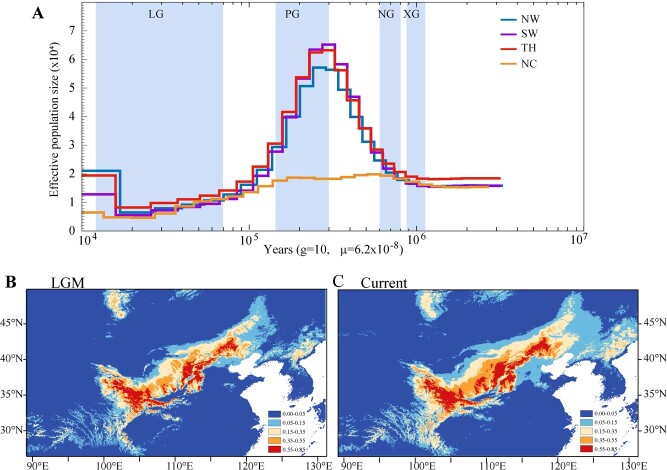
Demographic history and prediction of suitable habitats during the current time and the LGM. **A** PSMC for demographic history in four *P. cathayana* groups. Effective population size (*N*_e_) was inferred using the PSMC model. Four periods, the last glacial (LG, 11–70 kya), penultimate glaciation (PG, 130–300 kya), Naynayxungla Glaciation (NG, 500–780 kya), and Xixiabangma Glaciation (XG, 800–1170 kya), are shaded in blue. **B**, **C** Environmental niche modeling (ENMs) with Maxent predicted palaeodistributions and current distributions for *P. cathayana*: habitats that were predicted to be suitable at the LGM (**B**) and the current time predicted distribution (**C**).

The past climate history for *P. cathayana* had a profound effect on the species’ distribution and evolutionary history. We calculated the effective population size (*N*_e_) of *P. cathayana* from millions of years ago to the last 10 000 years. Three groups of *P. cathayana* (NW, SW, TH) exhibited concordant demographic trajectories, which reached the maximum *N*_e_ at ~200 kya, while *N*_e_ decreased continuously from the penultimate glaciation (PG) to the last glaciation (LG) (200–20 kya) (Fig. 4A). The NC group maintained a lower *N*_e_ and decreased slowly during the PG period. All four groups experienced a drastic contraction in the recent 100 kya and showed relatively strong positive Tajima’s *D* values, which indicated that a population bottleneck and balancing selection occurred.

When the present species distribution with environmental niche models (ENMs) was projected onto the Last Glacial Maximum (LGM) climate conditions, the predicted distribution showed a significant contraction for *P. cathayana* compared with its current distribution (Fig. 4B and C). The distribution of *P. cathayana* population contracted during the LG, which might have existence of glacial refuges. The demographic trajectories and species distribution support that this species experienced severe Quaternary climate shifts and associated environmental changes and our speculation about a very strong bottleneck during the history of *P. cathayana*.

### Genome-wide selective sweep signals of four *P. cathayana* groups

**Figure 5:**
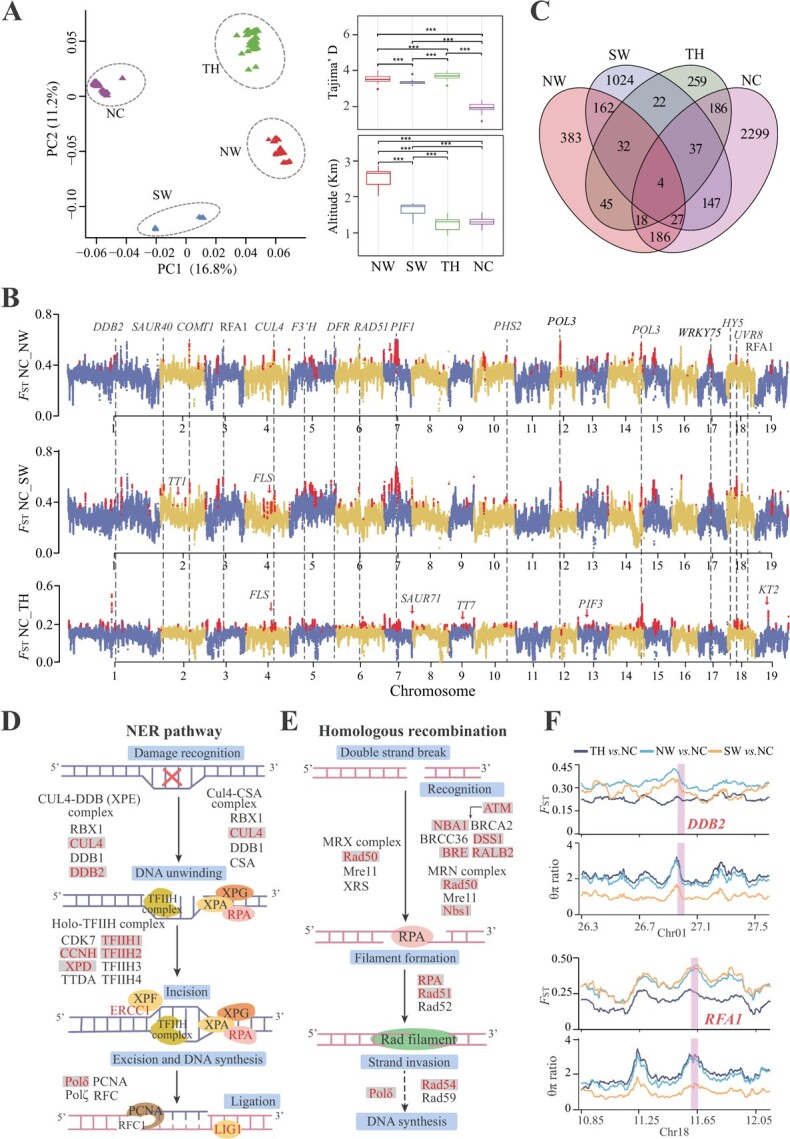
Population genetic differences and signatures of selective sweep. **A** PCA of selective sweep samples. Altitude and Tajima’s *D* have significant differences among the four groups. **B** Pairwise *F*_ST_ in selective sweeps for the compared groups. Each point represents one region. Other selective sweep regions are shown in Supplementary Data Fig. S7. **C** Venn diagram of genes in selective sweeps for the four compared groups. **D**, **E** Nucleotide excision repair (NER) and homologous recombination pathway enrichment distribution based on selective sweep genes. Represents the selected gene. **F** Two genes within regions having positive selection signatures, *DDB2* and *RAF1*, are visualized with shadows in line chart plots.

Four groups of *P. cathayana* with significant altitude differences had undergone long-term natural selection (Fig. 5A). According to the selective sweep results in NW, SW, TH, and NC, 713, 1455, 603, and 1760 candidate selection genes were identified combined with the top 5% of *F*_ST_ values and θπ ratios, respectively (Fig. 5B and C, Supplementary Data Fig. S7, Supplementary Data Table S20). Although few genes were shared by different groups, significant functional enrichment in the same terms was detected. Selective sweep genes were enriched in photosynthesis, defense response, DNA repair, and hormone biosynthesis and metabolic process by Gene Ontology (GO) and Kyoto Encyclopedia of Genes and Genomes (KEGG) annotations (Supplementary Data Fig. S8, Supplementary Data Table S21). These genes with strong selection signals were viewed as the foundation for local adaptation of *P. cathayana* in heterogeneous environments.

**Figure 6:**
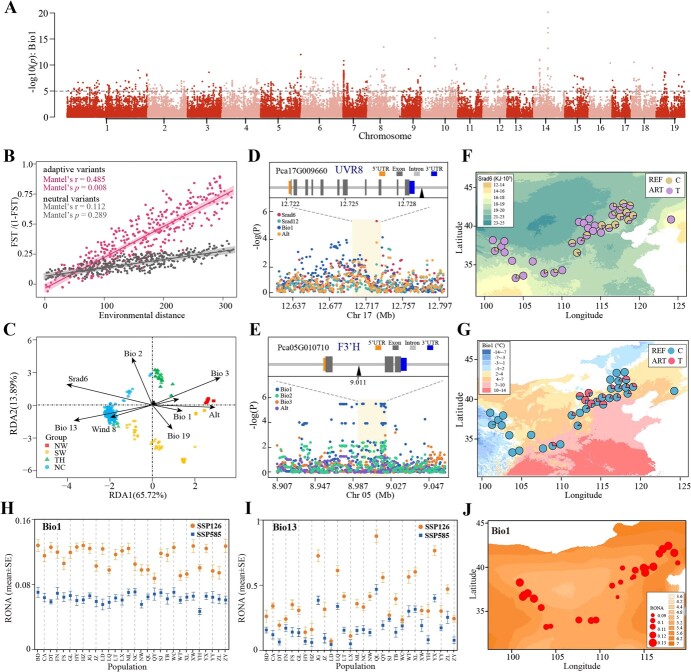
Environmental adaptation variables and response to future climate conditions in the *P. cathayana* population. **A** Manhattan plot for outlier loci associated with the Bio1 (annual mean temperature) variable. **B** IBE analyses for populations (*n* = 438) based on neutral variants and adaptive variants. The shadow of the linear regression represents the 95% confidence interval. **C** Partial RDA reveals the relationships between the independent environmental factors and population structure of *P. cathayana* along the RDA1 and RDA2 axes. Individuals are color-coded based on the four populations (NW, SW, TH, and NC). Vectors represent environmental variables. **D, E** Upper panels show the gene structure of *UVR8* (**D**) and *F3′H* (**E**) (triangles: candidate adaptive SNPs related to Srad6 or Bio1). Lower panels show local magnification of the Manhattan plots with environmental variables around the selected genes. **F, G** Candidate adaptive SNP allele frequencies (**F**, Chr17: 12725631; **G**, Chr05: 9005657) associated with Srad6 or Bio1 were investigated across the 36 populations. Color mapping is based on variations observed in the relevant range of environmental variables. **H**, **I** Comparison of average RONA values under two different climate scenarios (SSP126 and SSP585) in 2081–2100 across populations for Bio1 (*n* = 877 LFMM variants, *P* < .001) (**H**) and Bio13 (*n* = 1565 LFMM variants, *P* < .001) (**I**). Error bars represent the standard error of average RONA calculated from four different climate models. **J** Average RONA estimates of Bio1 across four climate models for the 24 populations under the SSP585 climate scenarios in 2081–2100. The raster colors on the map represent the degree of projected future climate change (absolute change). Areas with darker color are predicted to experience more substantial change in the respective climate variables. The size of the circles on the map represents the RONA values of different natural populations.

Most notably, the selection on DNA repair and photomorphogenesis genes may represent a major strategy for providing tolerance to UV irradiation damage in *P. cathayana* at high altitude. The enriched KEGG pathways under selective sweep contained nucleotide excision repair (NER), DNA mismatch repair (MMR), and homologous recombination (HR) (Supplementary Data Table S21). REPLICATION PROTEIN A1/2 protein (RPA1/2), XPE complex (*CUL4* and *DDB2*), and homo-TFIIH complex (*TF2H1*, *TF2H2*, and *XPD*) were noted for nucleotide excision repair (Fig. 5D), with *DDB2* and *RPA1* exhibited low nucleotide diversity and high genetic differentiation between NW and other groups (Fig 5F). In addition, MRN complex (*RAD50*), *RAD51*, *NBA1*, and *BRCA2* were found to participate in homologous recombination (Fig. 5E). The HYPOCOTYL 5 (*HY5*) gene associated with photomorphogenesis was located at the center of the protein–protein interaction (PPI) network hub resulting from selective sweep, which acted on genes related to DNA repair and response to light stimulus process (Supplementary Data Fig. S9). In addition, three genes (*DDB2*, *DCAF1*, and *CUL4*) were associated with *HY5* stability, and also exhibited a strong selection signal. Some key transcription factors (*bHLH*, *PIF3*, *APRR5*, *ARR2*) associated with *HY5* were under positive selection (Supplementary Data Fig. S9).

### Genomic variants associated with environmental adaptation

Local adaptation has the potential to create correlations between genomic loci and environmental variables (EVs). Nine EVs (Biol, Bio2, Bio3, Bio13, Bio19, Srad6, Srad12, Vapr6, and altitude) related to local adaptation were used for GEA analysis. These variables represent the extremes and seasonality of temperature, precipitation, and radiation pressure (Supplementary Data Table S22, Supplementary Data Fig. S10). First, 5773 environmental association loci (EALs) were identified as significantly associated with one or more EVs by use of the latent factor mixed model (LFMM); they were found not to cluster in specific genomic regions but to be widely distributed throughout the whole (Fig. 6A, Supplementary Data Fig. S11). Also, the strong and significant isolation by environment (IBE) observed in the adaptive variants by the Mantel test indicated that the adaptive genetic variants were mainly influenced by the environment (Fig. 6B). Next, 947 EALs associated with multiple environmental variables were identified using partial redundancy analysis (RDA) (Fig. 6C, Supplementary Data Fig. S12, Supplementary Data Table S23). Among the EALs in RDA, 11.62% were non-synonymous and 6.44% were synonymous mutations, and the non-synonymous rate showed no significant difference from all outlier loci (two-proportion *Z*-test, *P* = .267) (Supplementary Data Table S24). In addition, variation ratios of the 5′UTR (4.86%) and 3′UTR (7.50%) were significantly higher than those in the outlier loci (two-proportion *Z*-test, *P* < .001), indicated that environmental adaptation might mainly rely on selection through regulatory rather than protein-coding changes (Supplementary Data Table S24).

The key EALs for RDA were annotated to 391 genes (Supplementary Data Table S25, Supplementary Data Table S26). Among them, UV RESISTANCE LOCUS 8 (*UVR8*) was a photoreceptor mediating photomorphogenic responses to UV-B, and essential for photomorphogenesis and UV tolerance. To highlight the distribution pattern of allele frequencies, we focused on one essential adaptive SNP located downstream of *UVR8* (Pca17G009660) as an example (Fig. 6D). The T allele was mainly distributed in the NW China group, where solar radiation was high, whereas the C allele was more common in N and SW regions with weak solar radiation (Fig. 6E). Moreover, we found that a set of key UV irradiation genes, including cullin-4 gene (*CUL4*, Pca04G012380), DNA ligase 1 (*LIG1*, Pca04G008510), and Su(var)3–9 homologs (*SUVH4*, Pca05G019100), showed similar geographic distribution in frequencies (Supplementary Data Fig. S13). We identified multiple loci in temperature-related genes (Bio1, Bio2, and Bio3). For instance, the three tandem-duplicated genes encoding flavonoid 3′-hydroxylase (*F3*′*H*: Pca05G010730, Pca05G010700, and Pca05G010710) were significantly associated with Bio1 (Fig. 6F). A representative SNP variation was located in the intron of *F3*′*H*, which carried the T allele more frequently in high-altitude areas with lower temperature (Fig. 6G). The 75 genes identified in the GEA analysis were found within the genetic differentiation region, providing evidence of local adaptation in the *P. cathayana* population (Supplementary Data Fig. S14, Supplementary Data Table S27). The adaptive genes for selective sweep and GEA analysis were enriched in DNA repair and UV radiation response, such as *UVR8*, *HY5*, and *CUL4*. This finding emphasizes the role of these genes in high-altitude adaptation.

Using EALs by LFMM, we assessed the potential spatial pattern of maladaptation for *P. cathayana* using the risk of non-adaptedness (RONA). The values of RONA across the three future climate models were highly correlated across populations (Supplementary Data Fig. S15A, Supplementary Data Table S28). Average RONA values were calculated for each model, highlighting significant differences among populations (Fig. 6H and I). As expected, RONA increases under more severe climate change scenarios for most populations, as indicated by the comparison between SSP585 and SSP126 (Supplementary Data Fig. S15B–D). The temperature variable (Bio1) and precipitation variable (Bio13) indicated that the RONA value was stronger in areas with more drastic climate changes (Fig. 6J, Supplementary Data Fig. S15E–I). Considering climate change and the adaptive potential of variation, *P. cathayana* populations in North China will be confronted with maladaptation risks.

## Discussion

*Populus cathayana* is an important component of the *Tacamahaca* poplars. Using comprehensive sequencing technology, we have assembled the first high-quality *P. cathayana* genome, with 99.84% of the sequences ordered and orientated onto chromosomes. The favorable results of multiple evaluation approaches further confirm the high quality and completeness of assembly [27–29]. The WGD event provides original genetic material and plays a driving role in specialization and the creation of unique traits and functions [30]. Like other species of Salicaceae, *P. cathayana* underwent two WGD events, which led to the enrichment of gene duplications in defense and stimulation responses, and partially explained the expansion of the defense response-related gene family in the genome. In particular, we have discovered the expansion of disease resistance genes in *P. cathayana*, which may provide support for disease resistance breeding in poplar.

China has abundant germplasm resources of poplar belonging to the *Tacamahaca* section, mostly distributed in areas with variable terrain and a complex climate [1, 31, 32]. We present the first report on the collection and population genomic analyses of a large natural population of *P. cathayana* that is continuously distributed in China. Based on the population genetics, the NW group exhibits a broader genetic background, suggesting it as the genetic diversity and origin center of *P. cathayana*. Population structure can reflect past divergence events and gene flow [33–35]. Previous studies on *P. trichocarpa*, *P. davidiana*, and *P. deltoides* have shown that the demographic legacy of the Pleistocene climate change had a significant effect on genome-wide patterns of diversity [14, 36–38]. The *P. cathayana* population also has experienced bottleneck effects and contraction of its potential distribution range, as suggested by our ENM model, which may lead to the existence of glacier refuges in the eastern margin of the Qinghai–Tibet Plateau, the Qinling Mountains and North China. Some northern temperate plants in East Asia have hidden refugia at much higher latitudes than previously expected [35, 39–44]. The stable demographic history and variation haplotypes in the NC group suggest that ‘cryptic’ refugia exist and persist in poplar. We propose that the *P. cathayana* population has a complex history of large-scale populations affected by glaciers, and migration and diffusion from refugia after the ice age to form the present-day distribution.

The wild germplasm offers rich genetic resources that can be used to improve and enhance the growth characteristics and adaptability of trees [34, 45–47]. Through genome level analysis, the adaptive features of *P. cathayana* were studied. As observed in other widely distributed species [42, 48], the differences among *P. cathayana* lineages were highly heterogeneous in the genome. Additionally, GEA was employed to highlight loci with allele frequencies closely associated with environmental gradients [49, 50]. This rich information on the genetic basis of environmental adaptation may help in the development of effective strategies to mitigate the impact of climate change on species. Indeed, *P. cathayana* is primarily adapted to high altitude and grows near streams or ponds, where the genome is continuously challenged by intensive strong ultraviolet radiation and low temperature. Many previously reported genes, such as *HY5*, *CUL4*, and *RAD51* involved in UV-B signal transduction and DNA repair pathway, were prominent in the genome region associated with local adaptation [51–54]. This study identified hundreds of genes for local adaptation, many of which were functionally involved in the response to low temperature, providing support for low-temperature tolerance breeding [55, 56].

There are significant differences in RONA values among populations, indicated that adaptive differences in species stem from the genetic background and location. When considering the impact of future climate, it is necessary to consider not only the variability of allele frequencies, but also the climate risk of geographical location. Local adaptation provides only partial solutions at the genetic level for species to cope with the challenges of climate change. In summary, we conducted high-quality reference genome and population genome analysis of *P. cathayana*, which has enhanced our understanding of the genetic diversity of local poplar species in China. Given the context of genome-scale knowledge, we have the opportunity to address the challenge of addressing population vulnerability to future climate change [48, 53, 57, 58]. Our results can be used to help screen materials suitable for various environmental conditions from a large number of germplasms *in situ*, as they contain many unique and climate-adaptive genetic resources.

## Materials and methods

### Plant materials

A male *P. cathayana* individual was collected from Jiangou, Mentougou District, Beijing, China (116.05° E, 40.07° N, 922 m) for genome assembly. Tissue culture plants were produced using material from this individual. Young leaves, buds, and roots from the tissue-cultured seedlings were subsequently collected for RNA sequencing (Supplementary Data Methods S1). Next, a total of 438 wild germplasms of *P. cathayana* were collected for genome resequencing from 36 locations across its distribution range in China (Supplementary Data Table S16). Sampled individuals of each location were separated by at least 100 m. The geographic location information, including altitude, latitude, and longitude, of each individual was recorded using an Etrex GIS monitor (Garmin). All the above materials were preserved in the nursery of the Chinese Academy of Forestry in Beijing, China.

### DNA isolation and genome sequencing

The DNA used for Illumina sequencing and PacBio sequencing was extracted from leaves of tissue culture seedlings using a modified CTAB method. The Illumina library, with 270-bp insert size, was constructed following the manufacturer’s instructions and sequenced on the Illumina HiSeq X Ten platform. For PacBio library construction, genomic DNA was sheared to 20 kb and sequenced on the PacBio Sequel system. To satisfy the specific requirements of Hi-C sequencing, the fixed scheme was used to process and extract genomic DNA from leaf tissue, yielding DNA fragments ranging from 300 to 700 bp. The Hi-C library was sequenced on the Illumina HiSeq X Ten platform [59].

### Chromosome-level genome assembly

To obtain a high-quality genome assembly, the quality-controlled PacBio reads were initially corrected using a self-align method in NextDenovo software (v2.0) (https://github.com/Nextomics/NextDenovo). The corrected reads were then used to assemble the draft genome using the correction-before-assembly strategy. Indels and SNPs were subsequently corrected using Illumina sequencing data by Pilon software over three rounds [60]. The resulting assembly underwent further processing with purge_haplotigs to remove redundant haplotypes, producing a final contig-level assembly. The Hi-C data were utilized to further remove non-significant genome regions [61]. To refine the assembly, the clean reads were aligned to the contig-level assembly using Bowtie2 (v2.2.3) with default parameters. Using HiC-Pro (v2.10), the uniquely mapped paired-end reads from Hi-C data were retained for further analysis [62]. The uniquely mapped read pairs were used to clustered, ordered and orientated scaffolds onto chromosomes by LACHESIS [63]. Before chromosome assembly, scaffolds were divided into segments of ~50 kb. BWA software (v0.7.10-r789) was used to map the Hi-C data to these segments [64]. The placement and orientation that displayed distinct chromatin interaction patterns were corrected manually. To evaluate the final genome assembly, Illumina short reads were mapped to the genome using BWA (v0.7).

### Genome prediction and annotation

Firstly, three *de novo* prediction programs, RepeatModeler2 (v2.0.1) [65], RECON (v1.0.8) [66], and RepeatScout(v1.0.6) [67], were used to estimate the repeat composition in the genome (Supplementary Data Methods S1). Then, repeat sequences in this genome were classified using RepeatMasker (v19.06), REXdb (v3.0), Dfam (v3.2), and LTR_retriever (v2.8) [68–71]. The combined strategy of three approaches was used to predict protein-coding genes (Supplementary Data Methods S1). For the prediction of different types of non-coding RNA, tRNAscan-SE (v1.3.1) and miRBase (v21) were used to predict tRNA and miRNA with eukaryote parameters, respectively [72]. The rRNA genes were identified by Rfam (v12.0), and snoRNA and snRNA were predicted using INFERNAL against the Rfam (v1.1) [73, 74]. The qualities of the assembly and gene annotation were assessed using BUSCO (v5.2) and CEGMA (v2.5).

### Comparative genome analysis

Genome sequences from eight eudicot genomes of *P. trichocarpa*, *P. euphratica*, *P. alba**,** S. purpurea*, *Eucalyptus grandis*, *Quercus robur*, *Arabidopsis thaliana*, and *Oryza sativa* were obtained. The download addresses of the genomes are shown in Supplementary Data Table S14. OrthoFinder2 (v2.2.7) was used to identify orthologous genes among nine species [75]. The phylogenetic relationships were determined using PhyML (v3.1) based on single-copy orthologous genes, and the resulting phylogenetic tree was visualized using FigTree (v1.4.3) [76]. Divergence times were estimated using MCMCtree in PAML (v4.9i) [77]. To determine the expansion and contraction of gene families, the CAFÉ program (v3.1) was used to compare cluster size differences between ancestors and species [78]. 4DTV was used to evaluate the selection pressure and evolution rate of genome sequences, and PAML (v4.9i) software was used for calculation [77].

We utilized the BLAST (v2.12) tool to identify orthologous genes within the *P. cathayana* genomes (E-value <1e−5) [79] and MCScanX (python) was used to identify syntenic blocks and visualize the collinearity [80]. Using DupGen_finder with default parameters, we identified five duplicate types in the *P. cathayana* genome. The *K*_a_/*K*_s_ values of duplicate gene pairs were calculated using TBtools (v1.120) [81].

### Population resequencing and variation detection

Total genomic DNA from fresh and undamaged leaves was extracted using the modified CTAB method for resequencing. Paired-end sequencing libraries were constructed with an insert size of 150 bp. Whole-genome resequencing was conducted on the Illumina HiSeq 2500 platform, aiming for a target coverage of 30× per individual. After quality assessment using FastQC, low-quality bases with a Phred score <30 were trimmed from the reads using Trimmomatic. The high-quality paired-end sequencing reads were aligned to the *P. cathayana* genome assembly using the ‘mem’ algorithm of BWA software (v0.7.8). Duplicate reads were subsequently removed using SAMtools (v0.1.19) [82]. SNP calling was performed using GATK (v4.1.9.0) with the joint calling method [83]. We considered indel calls produced by SAMtools mpileup within a 1- to 50-bp window. For SV identification, BreakDancerMax (v1.4.4) was used to detect insertion (INS), deletion (DEL), and inversion (INV) [84]. We only retained variation supported by more than two read pairs. To detect CNVs, CNVnator (v0.3.2) with the parameter -call 100 was used [85]. ANNOVAR was utilized to annotate the genomic regions and quantities of SNPs, indels, CNVs, and SVs based on the *P. cathayana* reference genome with GFF3 files.

### Population structure and genetic diversity analysis

After variant calling SNPs were filtered with VCFTools [86] with parameters -min-meanDP 3 -minQ 30 -maf 0.05 -remove-indels -max-missing 0.2 -min-alleles 2 -max-alleles 2. A total of 12 374 210 SNPs were retained for further analysis. First, population structure was analyzed using ADMIXTURE (v1.23), and cross-validation error was tested for each cluster value (*K*) (2–15). Next, the TreeBeST program (v1.92) was employed to construct a neighbor-joining (NJ) tree. For PCA, GCTA (v1.24.2) was employed [87]. Homozygous individuals were retained for genetic diversity analysis, and grouping of individuals was shown in Supplementary Data Table S17. Pairwise genetic differentiation (*F*_ST_) was calculated using VCFtools (v0.1.16) with a window size of 100 kb and 1-kb steps to evaluate population differentiation [86]. The population recombination rate was estimated using FastEPRR, within a 100-kb window and 1-kb steps [88]. To assess LD decay, the LD coefficient (*r*^2^) between pairwise SNPs within a 500 kb window was calculated using PopLDdecay (v3.40) [89]. The Mantel test was executed between the *F*_ST_/(1 − *F*_ST_) matrix and geographical distance (km) matrix of the *P. cathayana* population using the R package vegan. Significance was determined based on 999 permutations. To calculate nucleotide diversity (π) and Tajima’s *D* value, VCFtools (v0.1.16) was utilized with a 100-kb sliding window for four groups [87].

### Demographic history and potential distribution prediction

PSMC (v0.6.5) was used to infer the history of effective population size (*N*_e_) of *P. cathayana* (μ = 6.2 × 10^−8^, *g* = 10 years) [90, 91]. Fastsimcoal2 (v2.1) was used to derive the best-fitting demographic model, estimate population differentiation time, and determine the per-generation migration rate of gene flow between clades [92]. TreeMix (v1.11) was used to model gene flow among subgroups of *P. cathayana* [93]. This inferred the maximum likelihood tree and identified potential gene flow based on the residual covariance matrix. Nine migration event models were established, and the optimal model was selected considering the AIC value and stability. Using the ENMs to predict the potential distribution areas of species, occurrence data were obtained from the sampling location of *P. cathayana*. The data for 19 bioclimatic variables during the current period (1970–2000) and the LGM were obtained from WorldClim (http://www.worldclim.org/) at a spatial resolution of 2.5 arcmin (~4.5 km). The bioclimatic data for the LGM were derived from the CCSM circulation model [94]. To calibrate and select the best ENMs, the Maxent algorithm implemented in the R package ENMeval was used [95, 96].

### Identification of selection sweep signatures

To detect selection signals during adaptation in the four groups, the admixed genotypes were first removed according to the population structure. A total of 358 samples of four groups were used to detect selective sweep signals [97]. Regions with signals for selective sweeps were identified using the top 5% *F*_ST_ values and the top 5% θπ ratios [25]. For the calculation of *F*_ST_, a window size of 100 kb and a step size of 1 kb were used. For the calculation of nucleotide diversity (π), a sliding window of 100 kb was employed. Genes located within selective sweep regions were considered as candidate genes. We performed GO and KEGG enrichment analysis for the candidate genes [98, 99]. The STRING database (v11.5) was also used to annotate homology genes and construct the PPI network [100].

### Collection of bioclimatic variable data

To assess the impact of environmental factors, we obtained environmental variables from WorldClim. These variables included 19 bioclimatic variables (Bio1–19), wind speed (Wind6 and Wind12), water vapor pressure (Vapr6 and Vapr12), and solar radiation (Srad6 and Srad12) for the period 1970–2000, with a resolution of 2.5 arcmin (Supplementary Data Fig. S21). These variables were extracted using DIVA-GIS (v10.6). The Pearson correlation between environmental variables was calculated, and variables with a correlation >0.8 were excluded, retaining nine environmental variables for further analysis. For each population, we downloaded future (2081–2100) data for the five EVs (Bio1, Bio2, Bio3, Bio13, and Bio19) from the WorldClim CMIP6 dataset of three climate models (BCCCSM2-MR model, ACCESS-CM2 model, and CMCC-ESM2 model; resolution 2.5 arcmin), with two shared socioeconomic pathways (SSPs): SSP126 and SSP585.

### Genotype–environment association analysis

Outlier loci were identified using BayeScan (v2.172) for GEA analysis, and the cutoff thresholds were a posterior probability >0.76 and a *q* value <0.05 [101]. The LFMM was used to test the relationship between outlier SNPs and nine environmental variables to determine the significant EALs [102, 103]. LFMMs were implemented by the R package Latent Environmental Association analysis (LEA), and natural genetic structure was incorporated as a random effect. SNP loci with |*z*| values ≥4 and *P*-values ≤1.0 e−5 associated with at least one environmental variable were considered as key EALs [102]. To investigate the role of genetic variation in adaptation (the key EALs from LFMM) and neutral variation (further LD pruned of SNPs), Mantel tests were separately used to test for associations between *F*_ST_/(*F*_ST_/1 − *F*_ST_) and environmental (IBE) distance, with significance determined using the R package vegan [7].

To explore the independent effects of environmental variables on environmental adaptability, the partial RDA model was conducted. The key adaptive EALs were used as response variables. Nine environmental variables were included as explanatory variables, while longitude and latitude were used as control variables to account for geographic factors. The RDA function in the R package vegan was used to perform partial RDA, and the anova.cca function was used to check the significance of environmental variables [104, 105]. The proportion of genetic variation explained by each RDA axis was calculated. SNPs that loaded on the tails of the distribution in RDA axes 1–3, with a cutoff of a 95% confidence interval, were considered as candidate loci and subjected to genomic annotation [105].

### Analysis of the adaptation potential of the genome to future climate change

Using the RONA approach, we calculated the theoretical allele frequency change needed to cope with future climate for EVs (Bio1, Bio2, Bio3, Bio13 and Bio19) [26]. The linear relationship between allele frequencies at significantly associated loci by LFMM and EVs was established using linear regressions (RONA parameter: LFMM, *P* < .001) [106, 107]. To avoid insufficient statistical efficacy or bias, 30 populations with more than six individuals were retained for RONA analysis. The difference (absolute value) between the present (1970–2000) and future (2081–2100, SSP585, BCC-CSM2-MR) was displayed on a map using Arcmap (v10.6).

## Acknowledgements

This work was supported by the National Key Research and Development Program of China (2021YFD2200201), the Major Project of Agricultural Biological Breeding (2022ZD04015), and the National Nonprofit Institute Research Grant of Chinese Academy of Forestry (CAFYBB2017ZY008).

## Author contributions

X.X., L.Z., and J.H. designed the project. D.C., Y.Z., L.Z., and J.H. collected the experimental materials. X.X. and J.H. performed the genome analyses. X.X., X.Z., H.W., and H.Z. performed resequencing and genetic analyses. X.X. and H.Z. contributed to the interpretation of results. X.X. wrote the manuscript. X.Z., L.Z. and J.H. revised the manuscript.

## Data availability

The *P. cathayana* genome and the sequencing data for genome assembly (Illumina reads, PacBio long reads, Hi-C reads, and RNA-seq data) have been deposited in the National Genomics Data Center (https://ngdc.cncb.ac.cn/?lang=en) under BioProject PRJCA014016. The whole-genome resequencing data have been deposited under National Genomics Data Center BioProject PRJCA014017 with the accession SAMC1029105-SAMC1029542.

## Conflict of interest

The authors declare no competing interests.

## Supplementary data

Supplementary data is available at *Horticulture Research* online.

## Supplementary Material

Web_Material_uhad255
