# Supplementary material for: Populus cathayana genome and population resequencing provide insights into its evolution and adaptation
Source: Hortic Res. 2023 Dec 11;11(1):uhad255. doi: 10.1093/hr/uhad255 (PMC10809908; doi:10.1093/hr/uhad255)
Supplement: Web_Material_uhad255 [file web_material_uhad255.zip › 2. Supplementary figure__202311.3.docx]

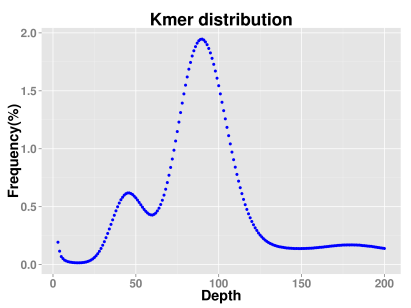


#### Figure S1 Genome size and heterozygosity estimation using 19 K-mer distribution.


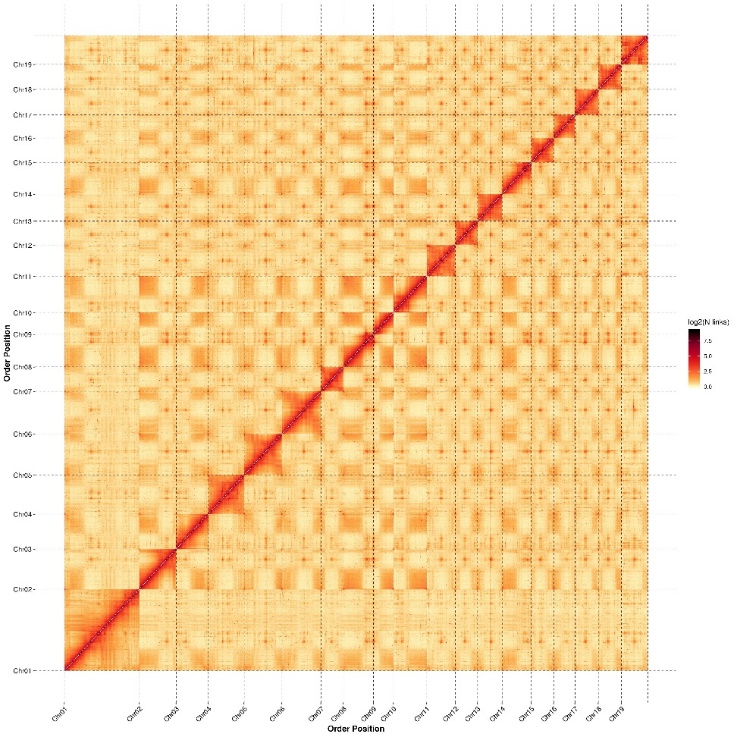


#### Figure S2 Mutogram between all chromosomes in the *P. cathayana* genome.

The interaction frequencies among 19 pseudo-chromosomes were drawn. The deeper the color, the higher the frequency of interaction.

**
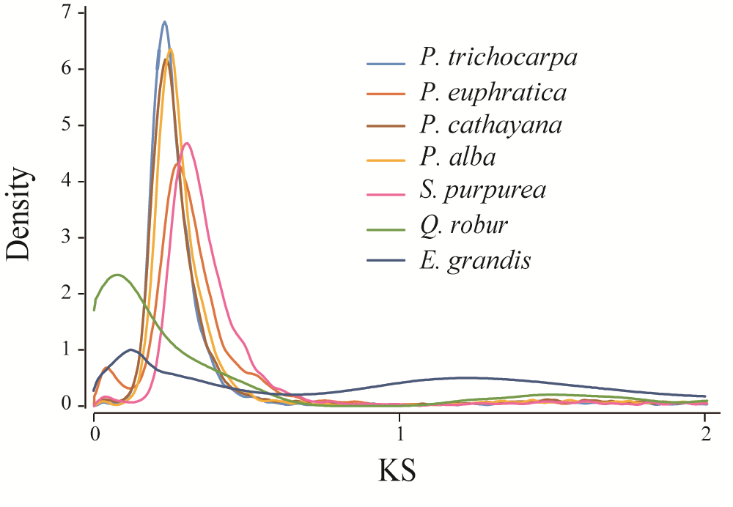
**

#### Figure S3 The KS values for paralogs in *P. euphratica*, *P. trichocarpa*, *P. alba*, *E. grandis, S. purpurea*, *Q. robur*, and *P. cathayana*.


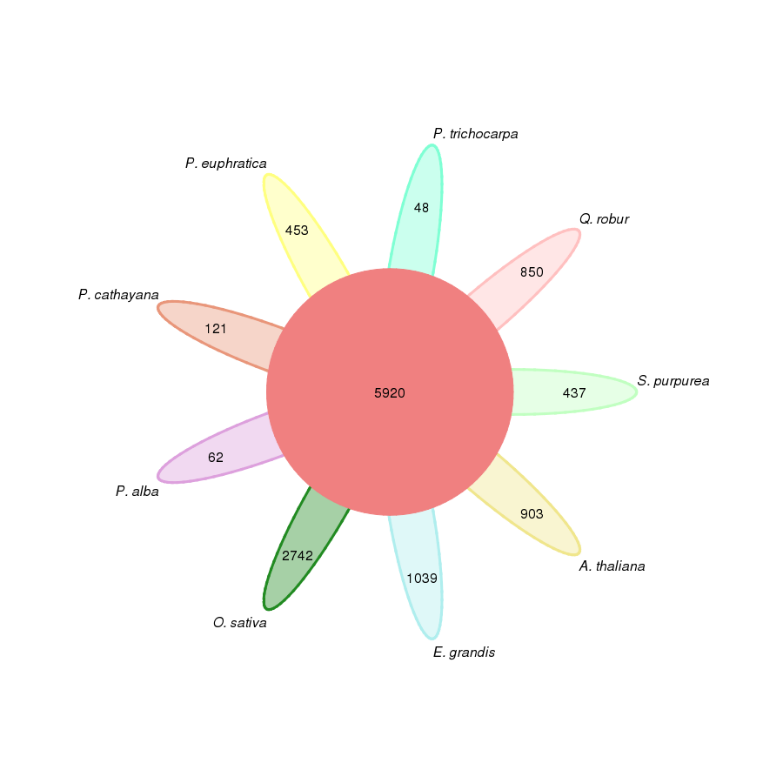


#### Figure S4 Number of gene families shared and unique in *P. cathayana*, *P. euphratica*, *P. trichocarpa*, *P. alba*, *E. grandis*, *S. purpurea*, *Q. robur*, *A. thaliana*, and *P. cathayana* species.


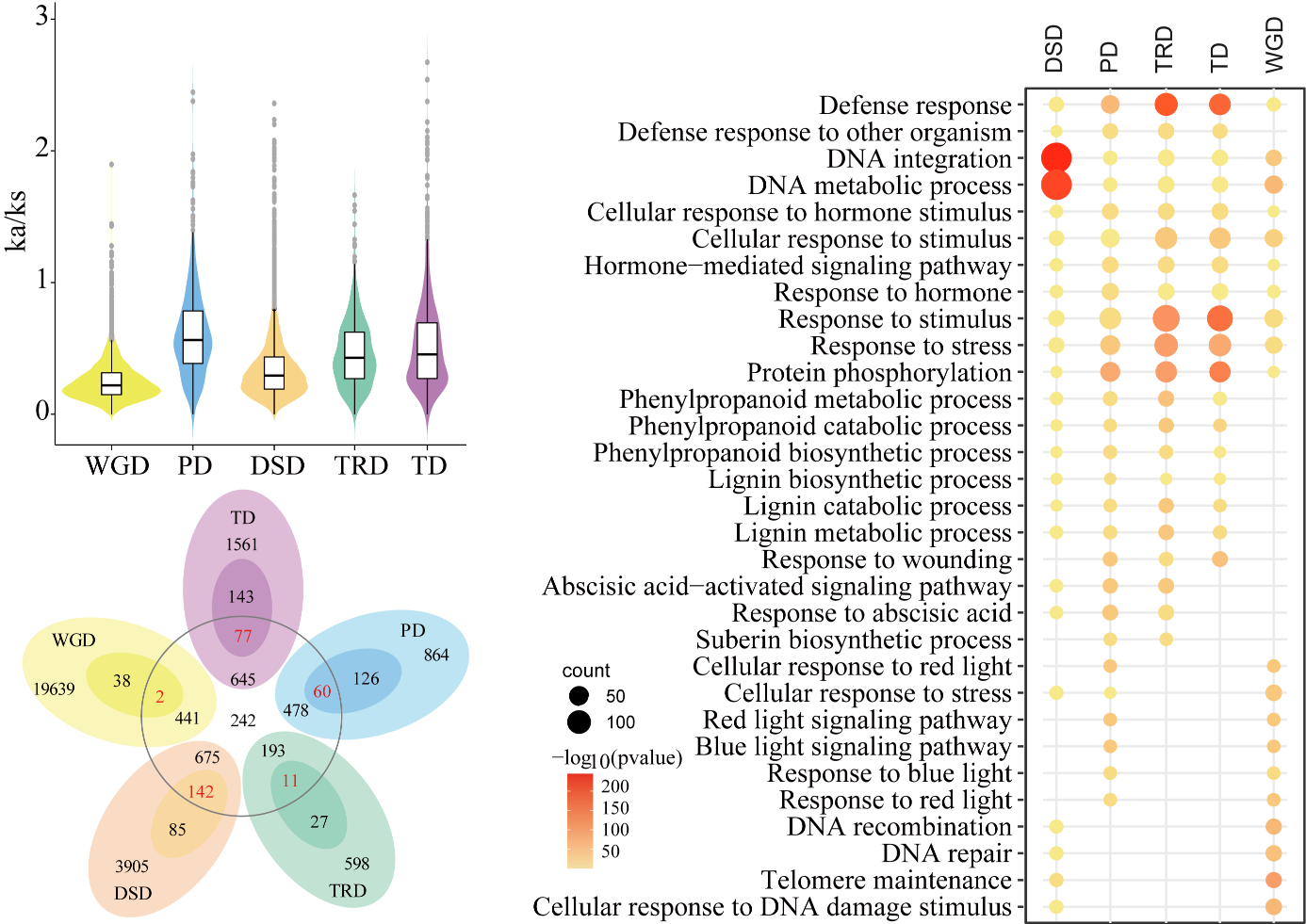


#### Figure S5 Functional enrichment of genes overlapping between expanded gene families (EGFs) and duplication genes.

The colors of the circles represent the statistical significance of enriched GO terms. The size of the circles represents the number of genes in GO term.


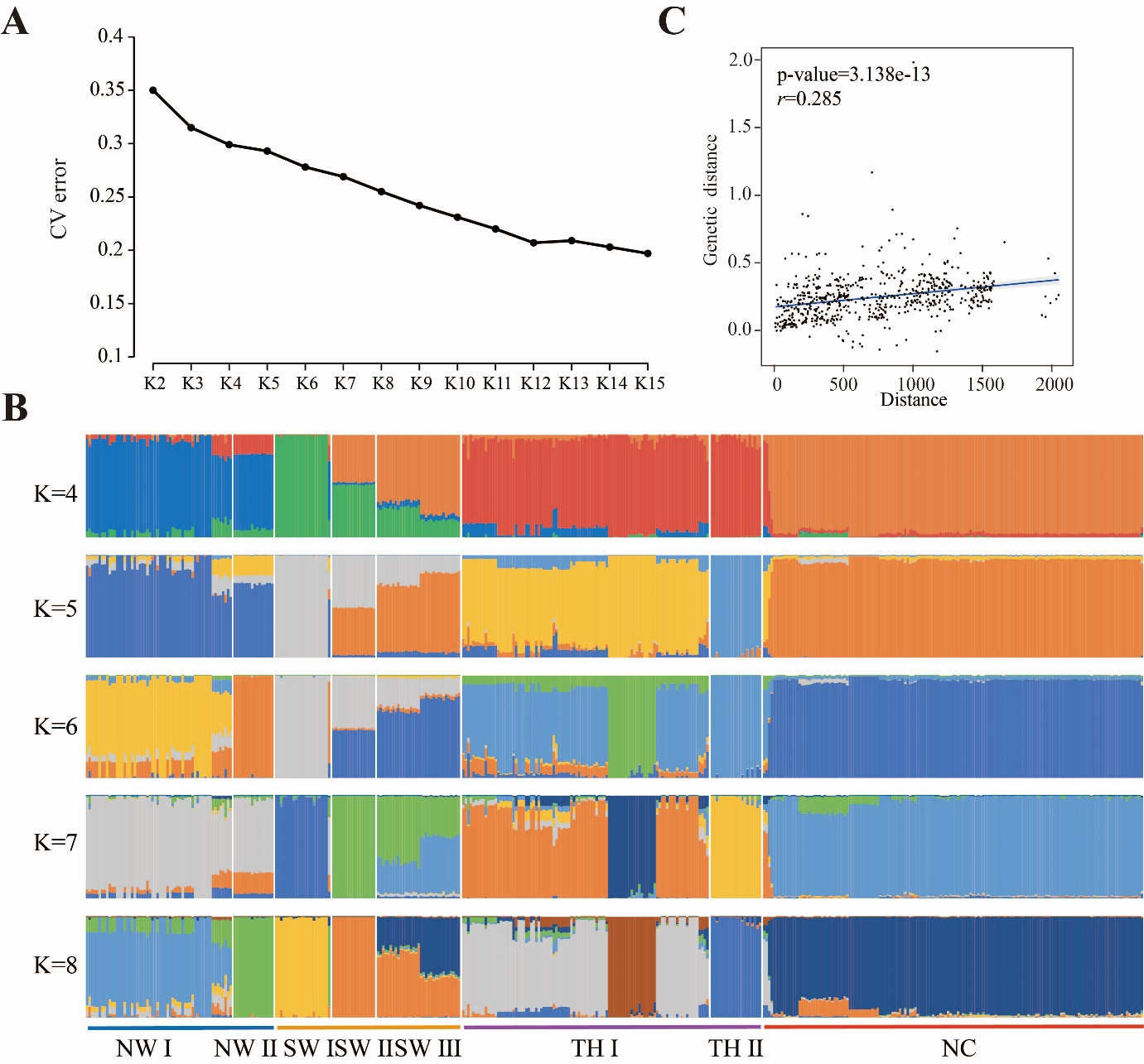


#### Figure S6 Results of the population structure and Mantel test in *P. cathayana* population.

(A). K (X-axis) represents the number of clusters considered. The Y-axis represents the cross-validation (CV) error rate of the model for different numbers of clusters. (B). Population genetic structure based on neutral SNPs estimated by ADMIXTURE analysis with K = 4 to 8. Some subgroups were formed at K > 4, thus we further investigated 4 groups as this clustering was consistent with the background of geographic distribution information of the population. (C). Relationships of genetic distance with geographical distance of *P. cathayana* populations using Mantel tests.


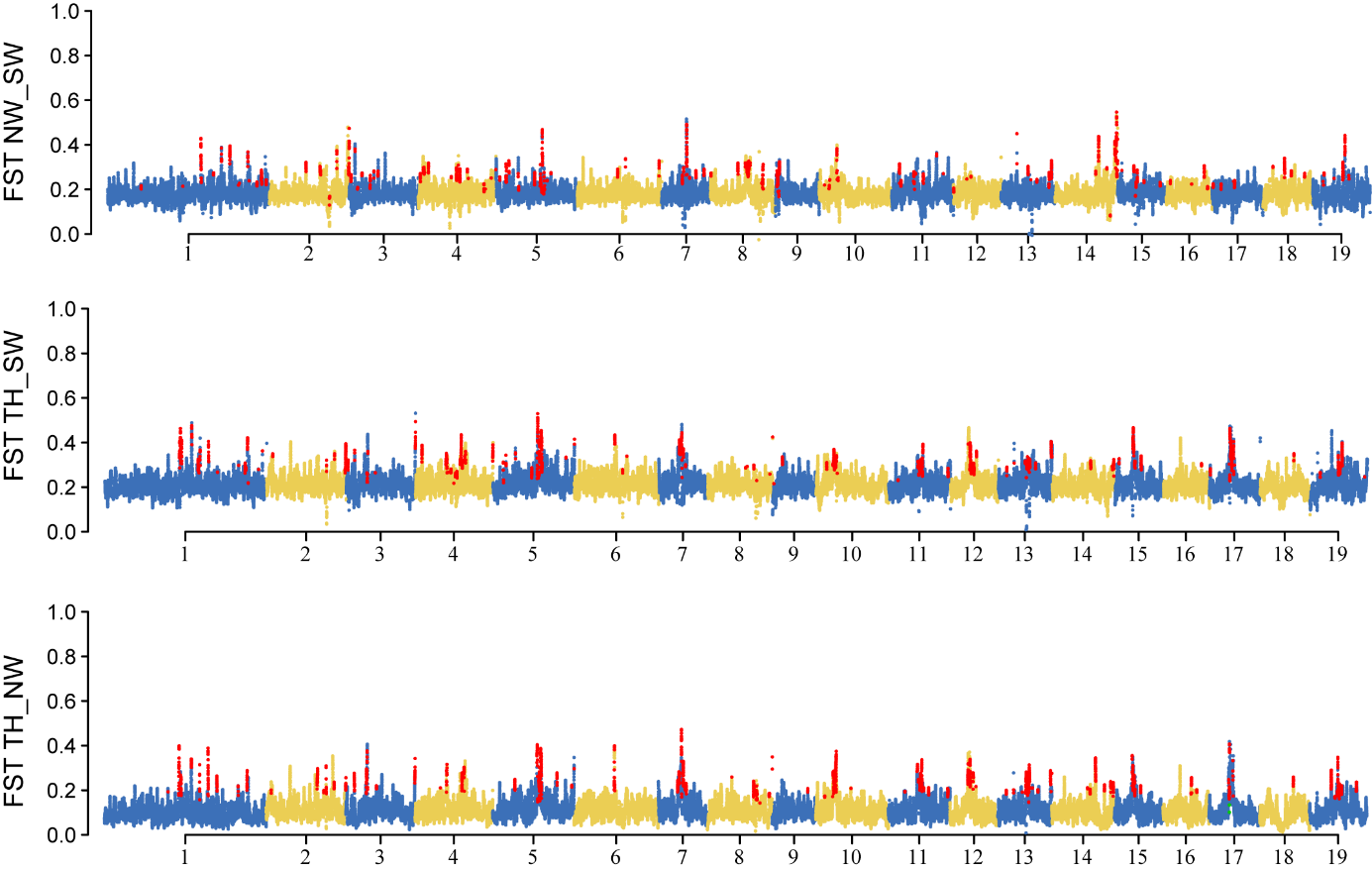


#### Figure S7 Selective sweep regions for population differentiation in *P. cathayana*.

The pairwise *F*_ST_ and *π* selective sweeps for the compared groups. Each point represents one region. The red spots represent genomic regions with the high *F*_ST_ and *π* ratio.


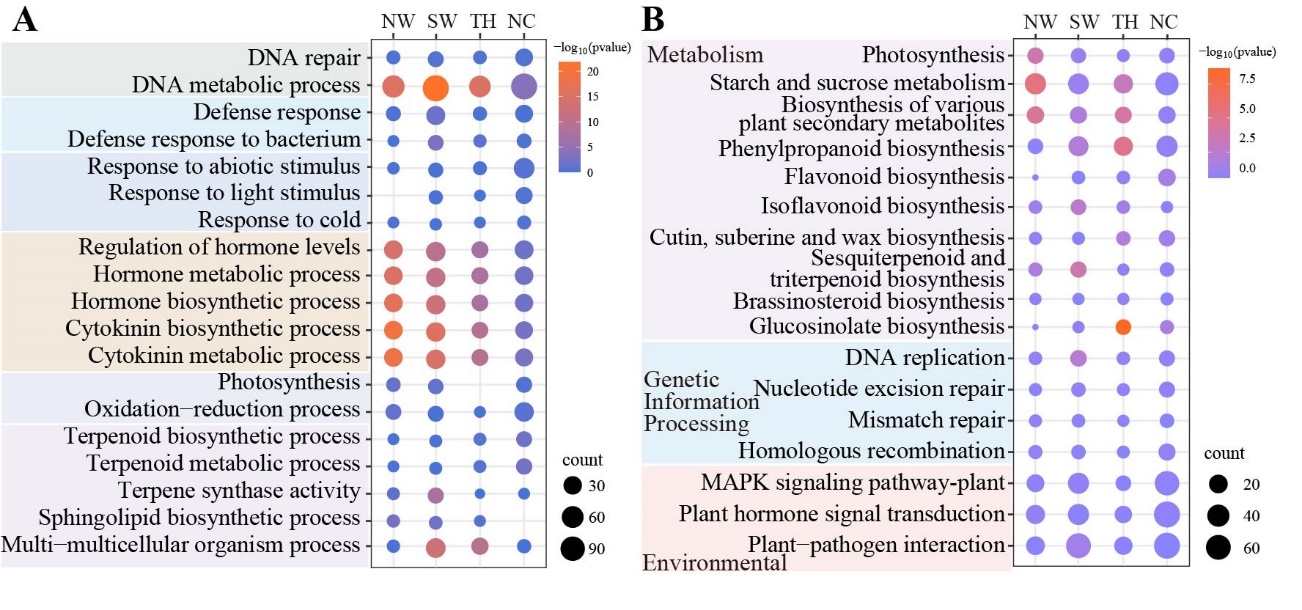


#### Figure S8 The GO and KEGG enrichment for selective sweep genes in four groups.

(A). GO enrichment for selective sweep genes. (B). KEGG enrichment for selective sweep genes.


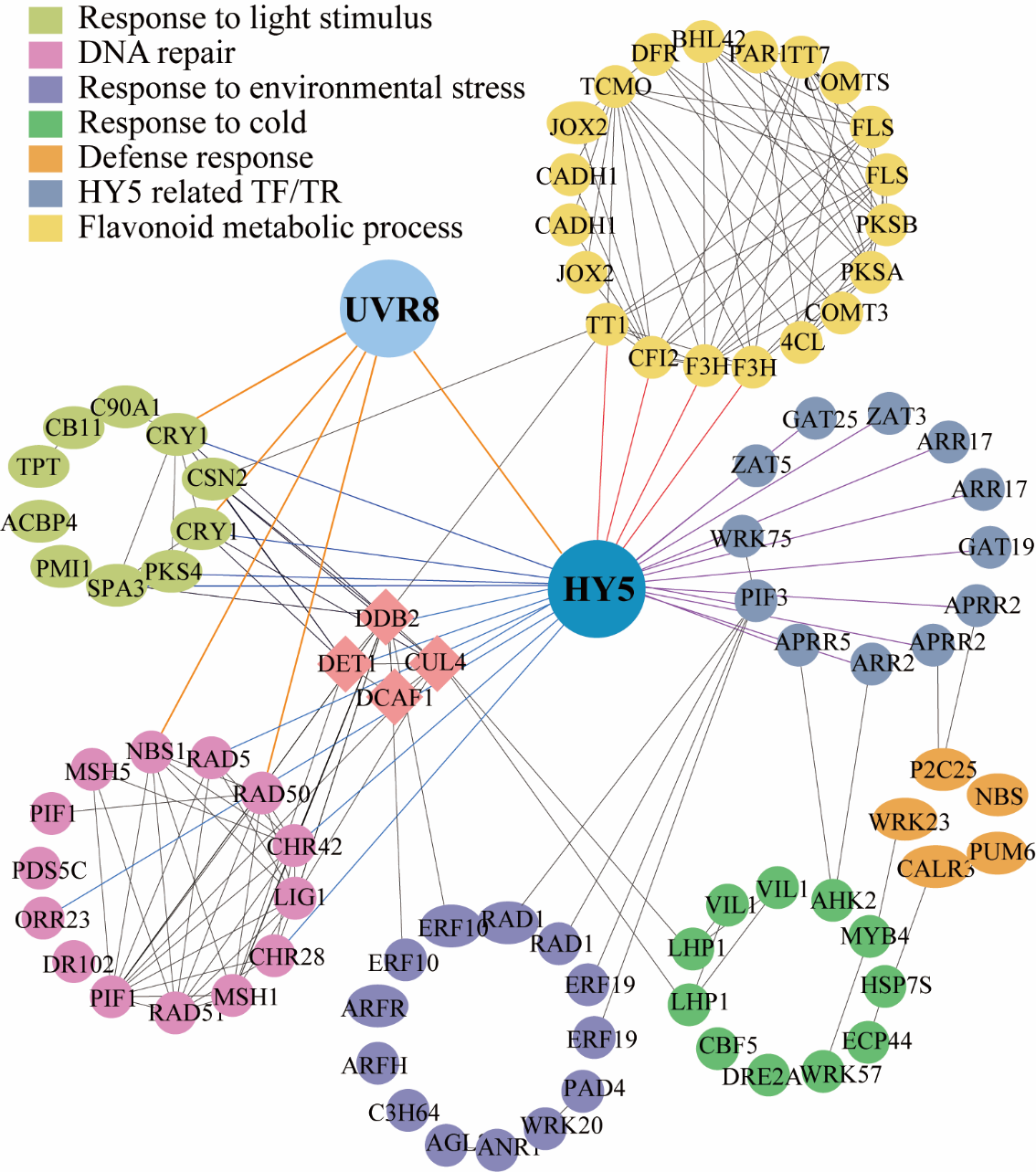


#### Figure S9. Core genes and gene regulatory network related to adaptation for selective sweeps in *P. cathayana*.

We annotated gene of local adaptation using the STRING database and created gene network diagram with HY5 as the core gene. Several selective sweep genes responsive to cold, response to light stimulus, DNA repair, response to environmental stress, defense response, and flavonoid metabolic process were shown.TF/TR: Transcription factors/Transcriptional regulatory factors.


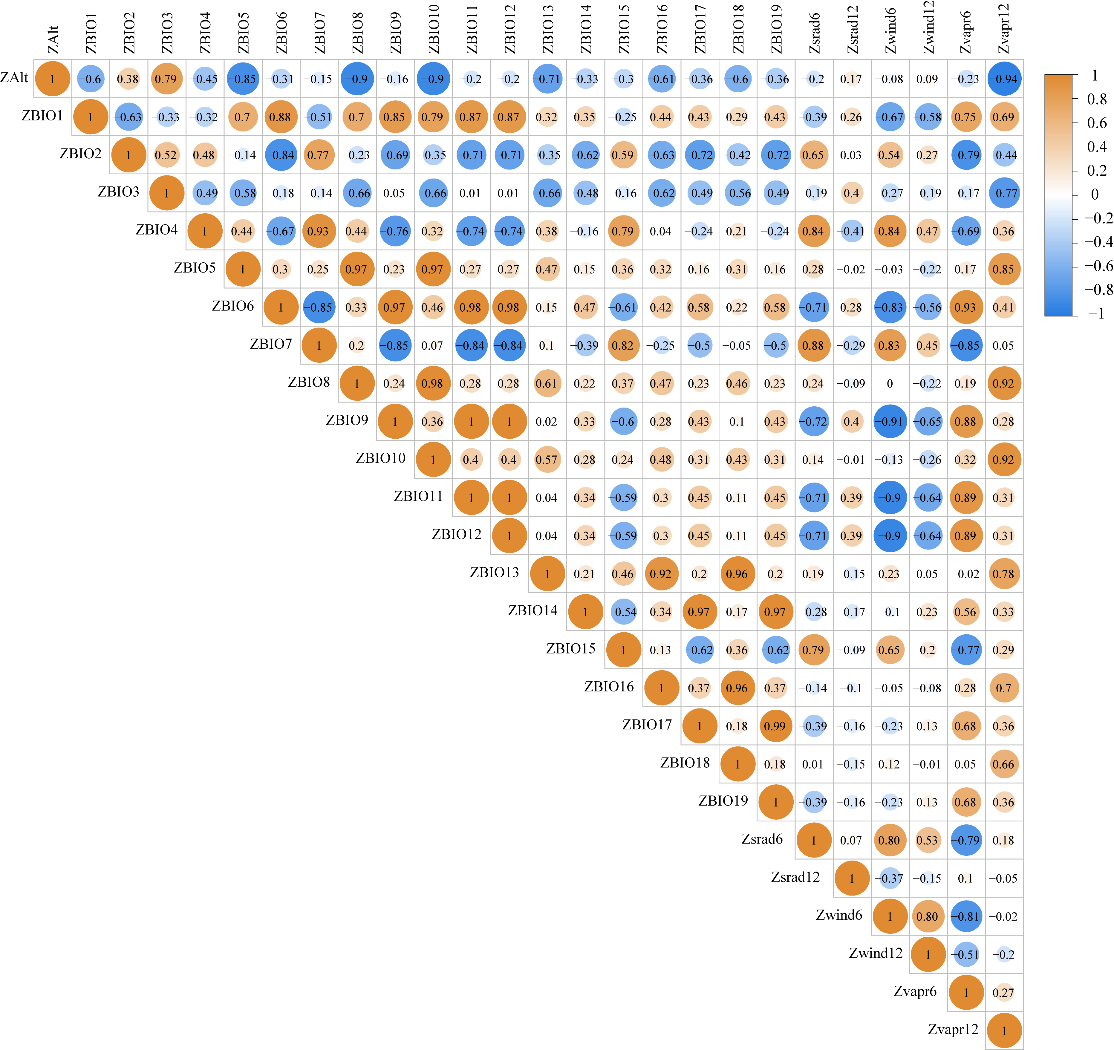


#### Figure S10. Pearson correlation coefficient (two-sided test) of 26 environmental variables.

After removing variables with a correlation higher than 0.8, retain Bio1, Bio2, Bio3, Bio13, Bio19, altitude, srad6, srad12, and vapr6 for subsequent analysis. Bio1: Annual mean temperature, Bio2: Mean diurnal range (mean of monthly (max temp - min temp)), Bio3: Isothermality (Bio2/Bio7) (*100), Bio13: Precipitation of Wettest Month, Bio19: Precipitation of coldest quarter, Vapr12: Water vapor pressure in December, Srad6: Solar radiation in June, Srad12: Solar radiation in December, Alt: Altitude.


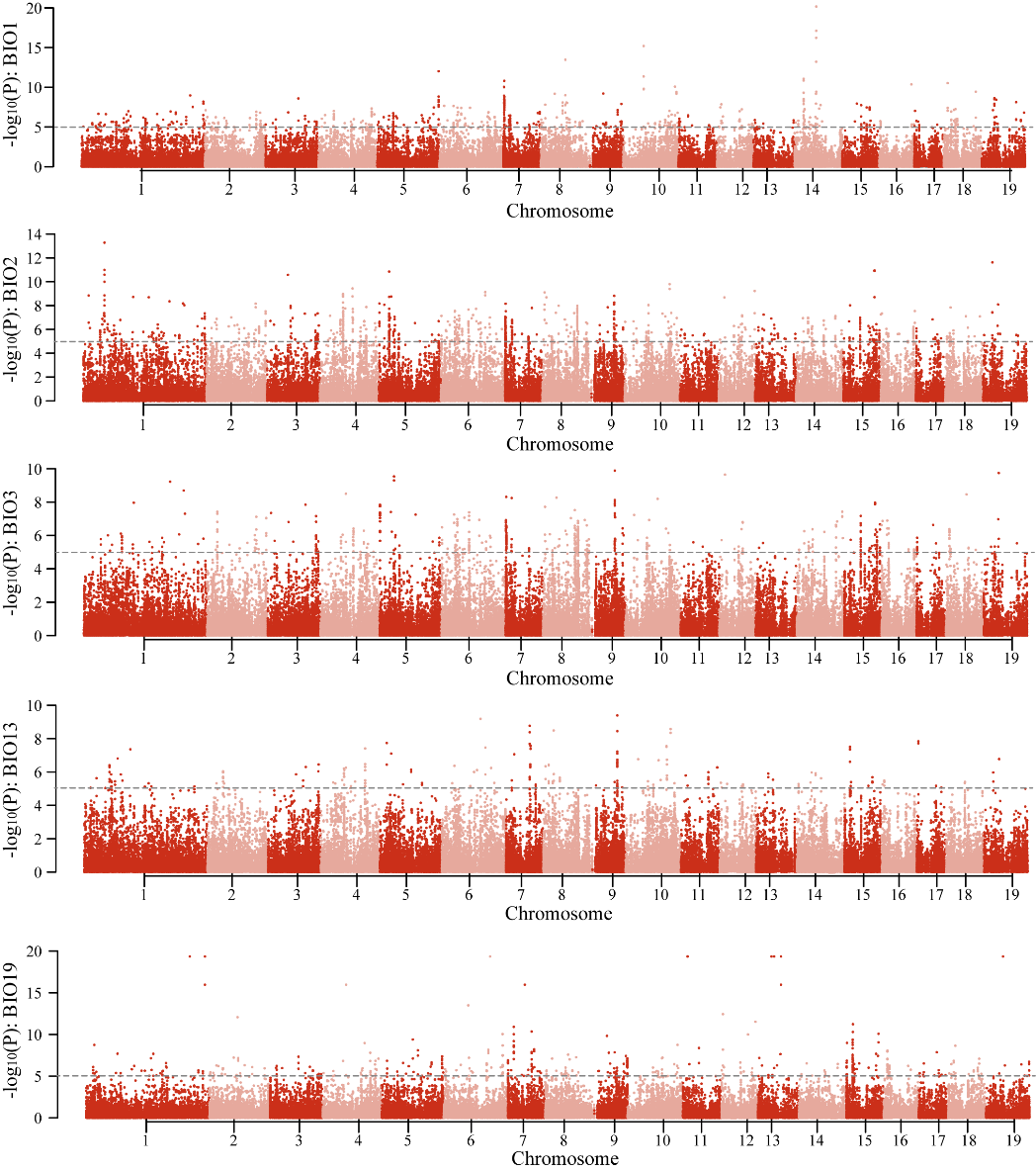

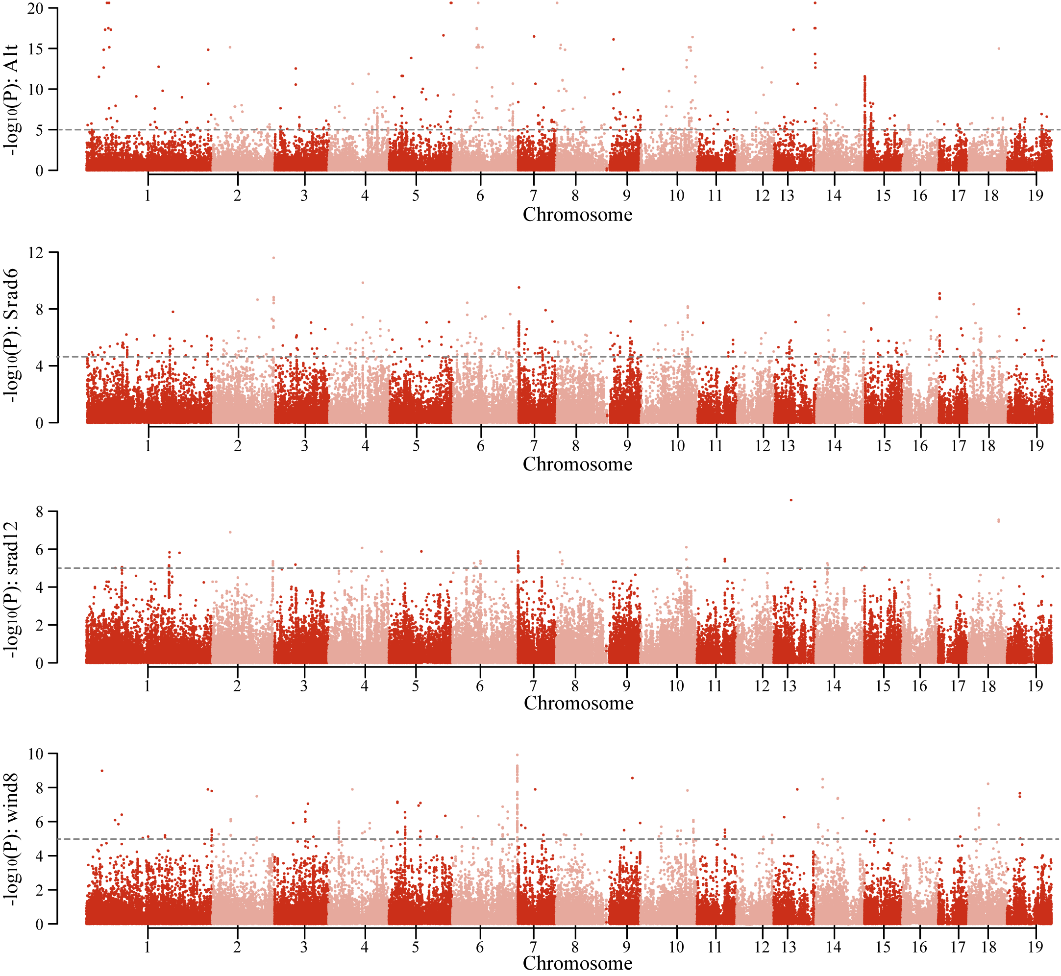


#### Figure S11 Manhattan plot for LFMM variants associated with the eight environmental variables.


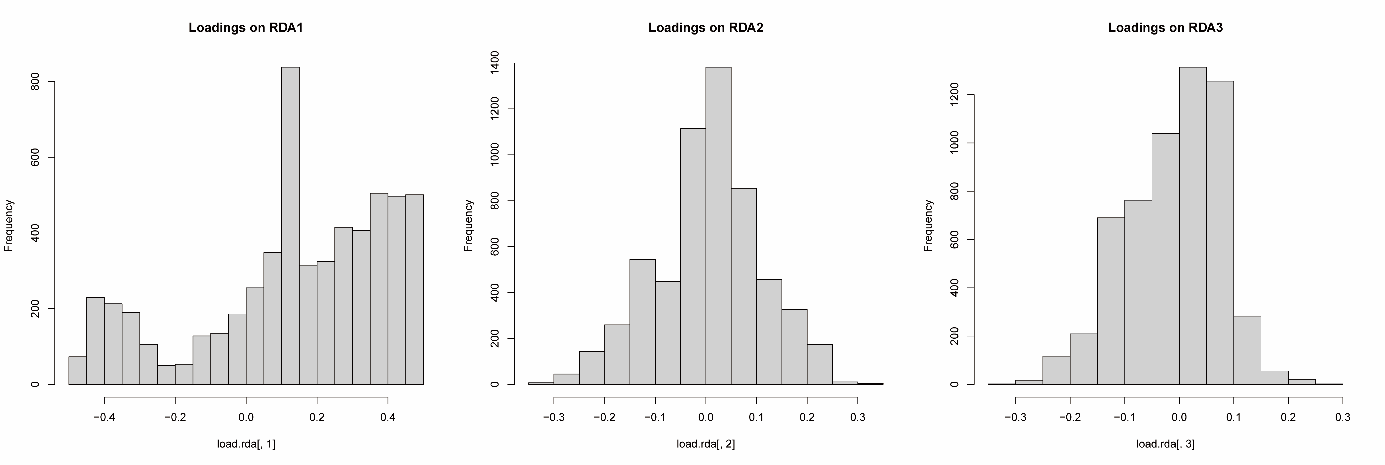


#### Figure S12 SNPs loading in RAD1, RAD2 and RAD3 by partial RDA analysis.

Histograms of SNPs loaded on the first three significant RDA axes. Null distributions are shown by hollow bars. SNPs loaded on the tails are more likely to be under selection as a function of the predictors.


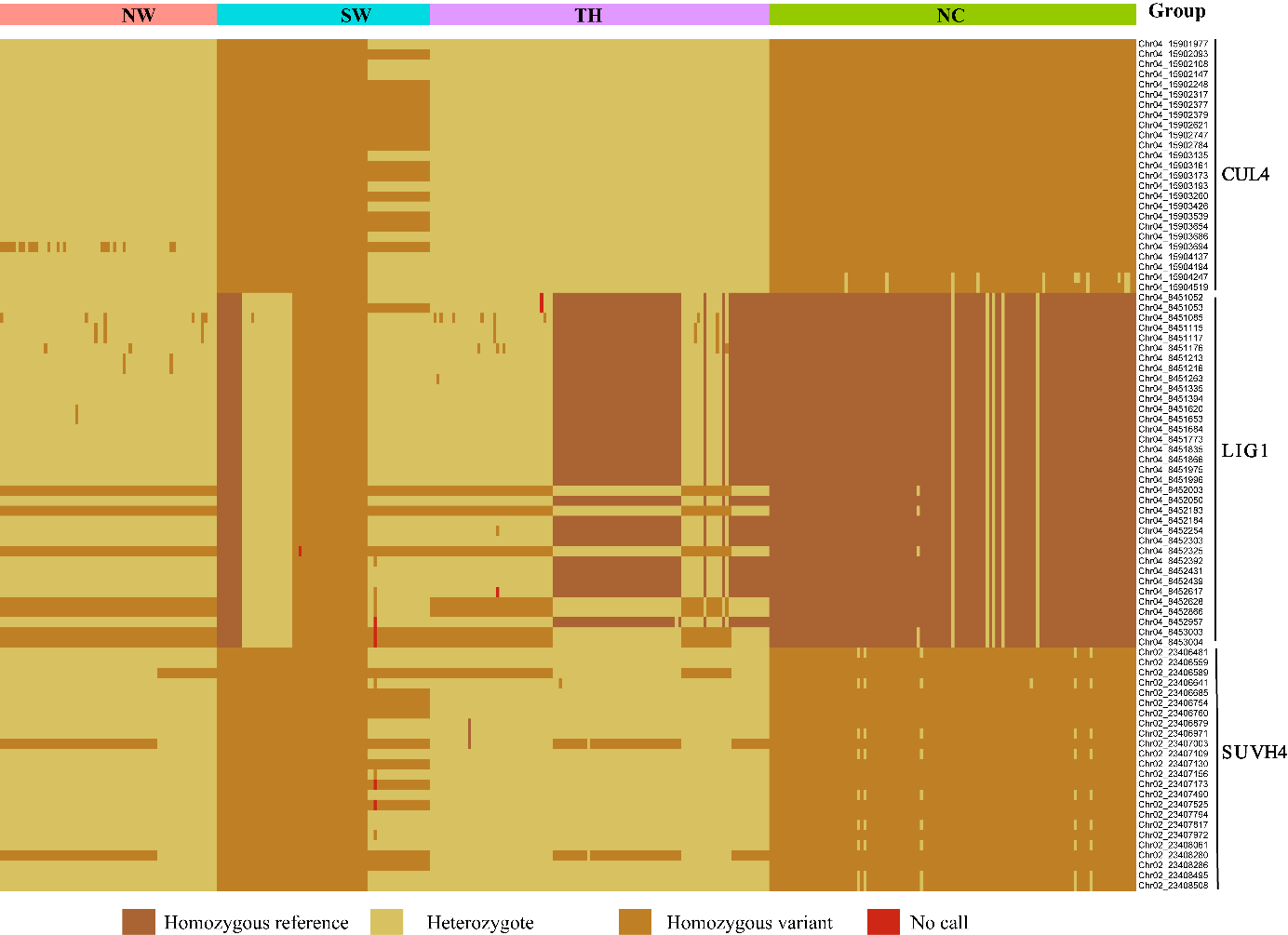


#### Figure S13 Haplotype differentiation patterns of adaption genes in *P. cathayana*.

Across the NW, SW, TH, and NC groups, significant haplotype differentiation patterns were observed for several SNPs within GEA candidate genes related to methyltransferase (*SUVH4*) and light signal (*CUL4* and *LIG1*).


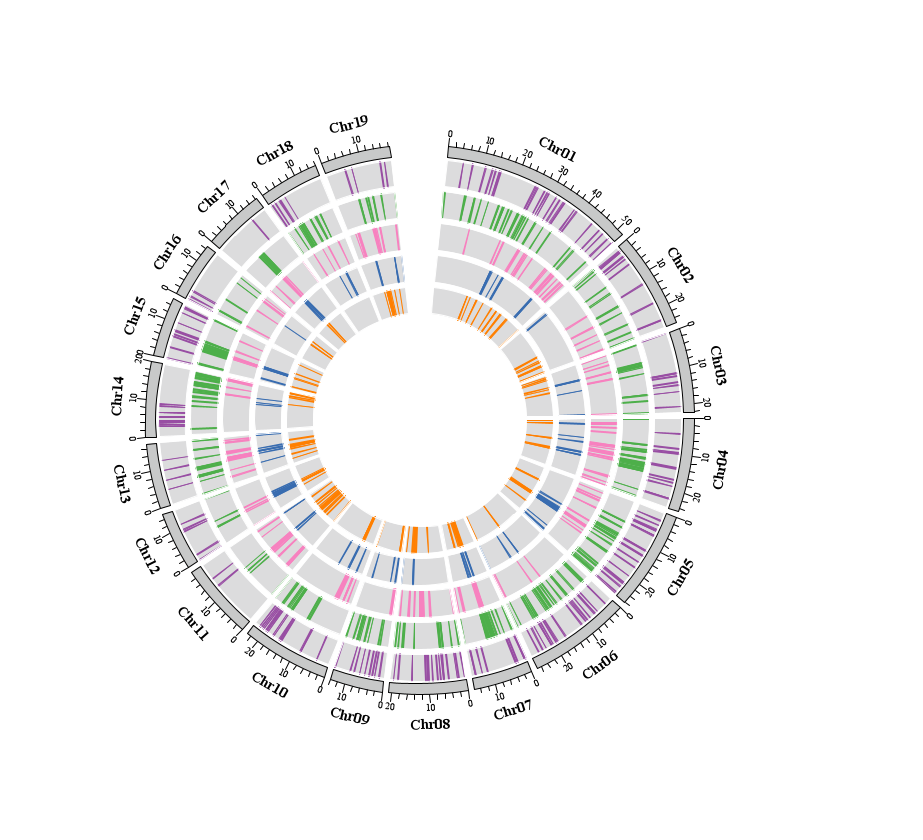


#### Figure S14 Circos map of the selective sweep regions and environmental association loci.

From the outside to the inside, the markers represent selective scanning regions in NW, SW, TH, and NC, with some genes annotated by RDA analysis at the innermost layer.


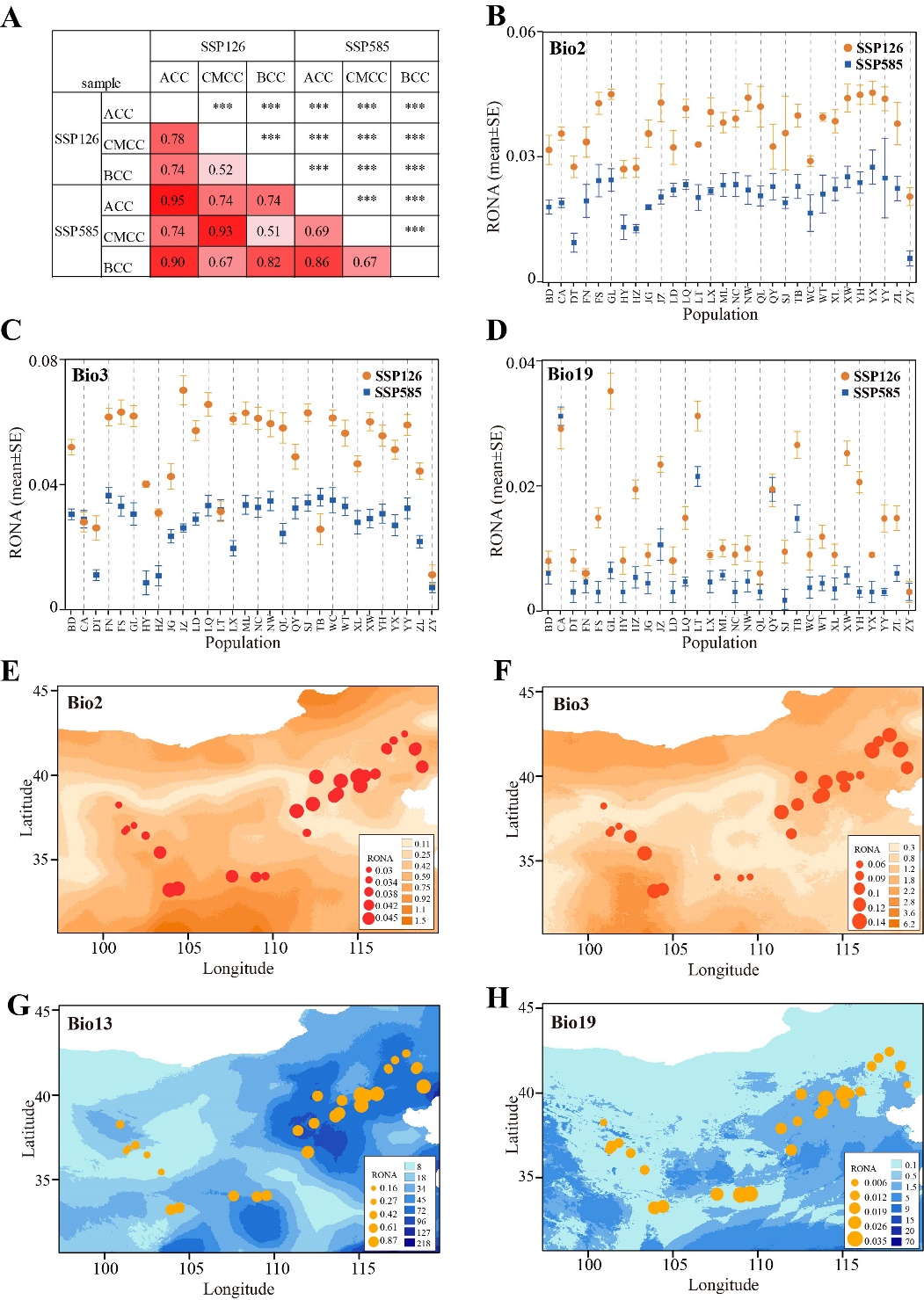


#### Figure S15 Risk of non-adaptedness (RONA) of P. cathayana to future climatic conditions.

(A), The correlations of the risk of non-adaptedness (RONA) values estimated among three future climate models using Spearman’s correlation analysis. Asterisks indicate significance levels (***P<0.001). BCC: BCC-CSM2-MR; ACCESS: ACCESS-CM2; CMCC: CMCC-ESM2. (B-D), The comparison of the average RONA values under two different climate scenarios (SSP126 and SS585) in 2081-2100 across populations for BIO2, BIO3, and BIO19 is shown. The average RONA estimates across three climate models for the 30 populations are presented, with error bars representing the standard deviation (SD) of the average RONA. (E-H), The raster colors on the map represent the degree of projected future climate change (absolute change) under BCC model with the SSP585 in 2081-2100, with darker areas experiencing more intense changes.
