# Supplementary material for: Populus cathayana genome and population resequencing provide insights into its evolution and adaptation
Source: Hortic Res. 2023 Dec 11;11(1):uhad255. doi: 10.1093/hr/uhad255 (PMC10809908; doi:10.1093/hr/uhad255)
Supplement: Web_Material_uhad255 [file web_material_uhad255.zip › 3. Supplementary tables_S1-S14 .docx]

Table S1 Statistics of sequencing data used for the P. cathayana genome assembly and annotation.

| **Libraries** | **Insert** | **Clean Reads Number** | **Clean data (Gb)** | **Sequence coverage (×)** |
| --- | --- | --- | --- | --- |
| PacBio reads | 20 Kb | 3,880,830 | 60.71 | 149.34 |
| Illumina reads | 270 bp | 158,096,009 | 47.35 | 116.47 |
| Hi-C reads | 350 bp | 169,353,078 | 50.63 | 124.53 |
| RNA sequence reads | 350 bp | 48,889,703 | 14.54 | 35.76 |

**Table S2** Length distribution of PacBio clean reads.

| **Length(bp)** | **Reads Number** | **Total length (bp)** | **Average length (bp)** | **Length ratio (%)** |
| --- | --- | --- | --- | --- |
| 2000~4000 | 465,077 | 1,364,957,492 | 2,935 | 2.25 |
| 4000~6000 | 369,923 | 1,839,858,658 | 4,974 | 3.03 |
| 6000~8000 | 321,936 | 2,246,980,058 | 6,980 | 3.70 |
| 8000~10000 | 288,645 | 2,592,418,601 | 8,981 | 4.27 |
| 10000~12000 | 272,125 | 2,993,122,729 | 10,999 | 4.93 |
| 12000~14000 | 289,242 | 3,763,796,606 | 13,013 | 6.20 |
| 14000~16000 | 294,099 | 4,408,024,496 | 14,988 | 7.26 |
| 16000~18000 | 259,610 | 4,406,171,916 | 16,972 | 7.26 |
| 18000~ | 1,320,173 | 37,096,005,837 | 28,099 | 61.10 |
| Total | 3,880,830 | 60,711,336,393 |  |  |

**Table S3** *P. cathayana* genome size from flow cytometry.

| **Species** | **Fluorescence intensity** | | | **Fluorescence intensity mean** | **Genome size (Mb)** |
| --- | --- | --- | --- | --- | --- |
| *P. cathayana* | 40.81 | 49.48 | 51.2 | 47.16 | 412.4 |
| *P. trichocarpa* | 44.84 | 43.73 | 46 | 44.86 | 392.3 |

* P. trichocarpa was used as internal standard. For the genome size of P. trichocarpa, refer to Hofmeister et al. Genome Biology (2020) 21:259.

**Table S4** The initial genome assembly.

| **Contig Num** | **Contig Len** | **Contig N50** | **Contig N90** | **Contig Max** | **GC(%)** |
| --- | --- | --- | --- | --- | --- |
| 74 | 411,495,660 | 10,281,541 | 3,296,020 | 23,331,369 | 34.20 |

**Table S5** Summary of contig/scaffold clustering results of final assembly in *P. cathayana*.

| **Final Assembly** | **Num** | **Len** | **N50** | **N90** | **Max** | **GC content(%)** |
| --- | --- | --- | --- | --- | --- | --- |
| Contigs stat | 77 | 406,544,207 | 10,281,541 | 3,296,020 | 23,331,369 | 33.84 |
| Scaffold stat | 21 | 406,549,807 | 20,860,933 | 15,353,775 | 51,967,413 |  |
| Gap stat | 56 | 5,600 |  |  |  |  |

**Table S6** Statistic of chromosomes of *P. cathayana*.

| **Chromosome id.** | **Cluster Contig Number** | **Cluster Sequence Length (bp)** | **Order Contig Number** | **Order Sequence Length (bp)** |
| --- | --- | --- | --- | --- |
| Chr01 | 10 | 51,966,513 | 10 | 51,966,513 |
| Chr02 | 3 | 25,587,339 | 3 | 25,587,339 |
| Chr03 | 10 | 22,236,917 | 10 | 22,236,917 |
| Chr04 | 5 | 24,925,147 | 5 | 24,925,147 |
| Chr05 | 4 | 26,189,997 | 4 | 26,189,997 |
| Chr06 | 7 | 27,300,257 | 6 | 27,117,915 |
| Chr07 | 4 | 15,752,662 | 4 | 15,752,662 |
| Chr08 | 5 | 20,860,533 | 5 | 20,860,533 |
| Chr09 | 2 | 14,021,428 | 2 | 14,021,428 |
| Chr10 | 1 | 23,331,369 | 1 | 23,331,369 |
| Chr11 | 3 | 19,921,237 | 3 | 19,921,237 |
| Chr12 | 1 | 15,353,775 | 1 | 15,353,775 |
| Chr13 | 4 | 17,346,366 | 4 | 17,346,366 |
| Chr14 | 4 | 20,573,503 | 3 | 20,110,857 |
| Chr15 | 1 | 15,667,764 | 1 | 15,667,764 |
| Chr16 | 5 | 14,745,476 | 5 | 14,745,476 |
| Chr17 | 3 | 16,333,218 | 3 | 16,333,218 |
| Chr18 | 2 | 16,015,902 | 2 | 16,015,902 |
| Chr19 | 3 | 18,414,804 | 3 | 18,414,804 |
| Total  (Ratio %) | 77(100.0) | 406544207(100.0) | 75(97.4) | 405899219(99.84) |

**Table S7** Illumina reads mapped to the genome.

| **Reads number** | **Mapped reads** | **Mapped (%)** | **Properly mapped reads** | **Properly_mapped (%)** |
| --- | --- | --- | --- | --- |
| 316,192,018 | 303,525,916 | 95.99 | 286,081,514 | 90.48 |

**Table S8** BUSCO and CEGMA evaluation of the P. cathayana assembled genome.

| **Genome evaluation** | ***P. cathayana* Gene** |
| --- | --- |
| Complete BUSCOs | 1581 (97.96%) |
| Complete and single-copy BUSCOs | 1330 (82.40%) |
| Complete and duplicated BUSCOs | 251 (15.55%) |
| Fragmented BUSCOs | 11 (0.68%) |
| Missing BUSCOs | 22 (1.36%) |
| Number of 458 CEG* present in assembly | 450 (98.25%) |
| Number of 248 highly conserved CEGs present | 236 (95.16%) |

**Table S9** Statistics of species gene information.

| **Species** | ***P. cathayana*** | ***A. thaliana*** | ***P. alba*** | ***P. deltoides*** | ***P. euphratica*** | ***P. trichocarpa*** |
| --- | --- | --- | --- | --- | --- | --- |
| Number of genes | 35,977 | 27,381 | 30,363 | 44,853 | 38,258 | 34,699 |
| Total gene length  (bp) | 123,911,560 | 60,368,916 | 123,948,736 | 136,044,577 | 132,155,196 | 126,544,386 |
| Average gene length  (bp) | 3444.19 | 2204.77 | 4082.23 | 3033.12 | 3454.32 | 3646.92 |
| ExonLen Length | 61,497,232 | 40,579,948 | 64,348,103 | 48,528,846 | 57,424,927 | 43,737,174 |
| Average exons Num  per mRNA | 1709.35 | 1482.05 | 2119.29 | 1081.95 | 1500.99 | 1260.47 |
| Average exons Num  per gene (bp) | 5.4 | 5.31 | 6.72 | 4.75 | 5.52 | 5.13 |
| Average CDS length  (bp) | 1317.21 | 1217.76 | 1390.74 | 1081.95 | 1133.3 | 1260.47 |
| Average CDS Num  per gene (bp) | 5.22 | 5.12 | 5.57 | 4.75 | 5.29 | 5.13 |
| Average intron length  (bp) | 1734.84 | 722.73 | 1962.94 | 1951.17 | 1953.32 | 2386.44 |
| Average introns in CDSs  per mRNA | 4.4 | 4.31 | 5.72 | 3.75 | 4.52 | 4.13 |

**Table S10** Functional annotation of predicted genes for *P. cathayana*.

| **Anno_Database** | **Annotated Number** | **Annotated Ratio (%)** |
| --- | --- | --- |
| GO_Annotation | 28,645 | 79.62 |
| KEGG_Annotation | 26,007 | 72.29 |
| KOG_Annotation | 18,775 | 52.19 |
| Pfam_Annotation | 29,863 | 83.01 |
| Swissprot_Annotation | 28,170 | 78.3 |
| TrEMBL_Annotation | 35,314 | 98.16 |
| eggNOG_Annotation | 29,321 | 81.5 |
| nr_Annotation | 34,980 | 97.23 |
| All Annotated | 35,366 | 98.3 |

**Table S11** Repetitive sequences number and length.

| **Type** | **Number** | **Length** | **Rate(%)** |
| --- | --- | --- | --- |
| ClassI:Retroelement | 183,934 | 99,453,100 | 24.46 |
| ClassI/DIRS | 5 | 237 | 0 |
| ClassI/LINE | 27,519 | 8,062,237 | 1.98 |
| ClassI/LTR/Caulimovirus | 461 | 567,046 | 0.14 |
| ClassI/LTR/Copia | 22,319 | 16,934,437 | 4.17 |
| ClassI/LTR/ERV | 1,047 | 71,805 | 0.02 |
| ClassI/LTR/Gypsy | 41,658 | 39,405,108 | 9.69 |
| ClassI/LTR/Ngaro | 356 | 21,496 | 0.01 |
| ClassI/LTR/Pao | 133 | 8,738 | 0 |
| ClassI/LTR/Unknown | 81,616 | 33,016,113 | 8.12 |
| ClassI/SINE | 8,820 | 1,365,883 | 0.34 |
| ClassII:DNA transposon | 163,225 | 51,421,779 | 12.65 |
| ClassII/CACTA | 9,301 | 9,214,885 | 2.27 |
| ClassII/Crypton | 69 | 3,415 | 0 |
| ClassII/Dada | 297 | 15,231 | 0 |
| ClassII/Ginger | 83 | 4,090 | 0 |
| ClassII/Helitron | 625 | 390,754 | 0.1 |
| ClassII/IS3EU | 195 | 10,330 | 0 |
| ClassII/Kolobok | 367 | 20,084 | 0 |
| ClassII/Maverick | 68 | 3,828 | 0 |
| ClassII/Merlin | 317 | 17,111 | 0 |
| ClassII/Mutator | 2,075 | 919,411 | 0.23 |
| ClassII/P | 149 | 7,868 | 0 |
| ClassII/PIF-Harbinger | 2,330 | 975,494 | 0.24 |
| ClassII/PiggyBac | 99 | 3,817 | 0 |
| ClassII/Tc1-Mariner | 295 | 41,564 | 0.01 |
| ClassII/Unknown | 142,179 | 38,358,338 | 9.44 |
| ClassII/Zisupton | 134 | 6,926 | 0 |
| ClassII/hAT | 4,642 | 1,428,633 | 0.35 |
| Unknown | 23 | 1,518 | 0 |
| Unspecified | 2 | 169 | 0 |
| Total | 347,184 | 150,876,566 | 37.11 |

**Table S12** Statistics of tandem repeat sequences.

| **Type** | **Number** | **Length** | **Rate(%)** |
| --- | --- | --- | --- |
| microsatellite(1-9 bp units) | 256,576 | 4,519,165 | 1.11 |
| minisatellite(10-99 bp units) | 80,858 | 7,409,215 | 1.82 |
| satellite(>=100 bp units) | 13,359 | 11,019,528 | 2.71 |
| Total | 350,793 | 22,947,908 | 5.64 |

**Table S13** Number of non-coding RNA in *P. cathayana*.

| **Non-coding RNA Type** | **Number** |
| --- | --- |
| rRNA | 451 |
| tRNA | 788 |
| miRNA | 1,149 |
| snRNA | 85 |
| snoRNA | 500 |

**Table S14** The genome downloaded website of the species for comparative genomic analysis.

| **Species** | **Website** |
| --- | --- |
| *Populus trichocarpa* | <https://data.jgi.doe.gov/refine-download/phytozome?organism=Ptrichocarpa&expanded=533)> |
| *Populus euphratica* | <ftp://ftp.ncbi.nlm.nih.gov/genomes/all/GCF/000/495/115/GCF_000495115.1_PopEup_1.0> |
| *Populus abla* | <https://www.ncbi.nlm.nih.gov/data-hub/taxonomy/43335/> |
| *Salix purpurea* | <https://phytozome-next.jgi.doe.gov/info/Spurpurea_v5_1> |
| *Eucalyptus grandis* | [https://phytozome.jgi.doe.gov/pz/portal.html#!info?alias=Org_Egrandis](https://phytozome.jgi.doe.gov/pz/portal.html#!info?alias=Org_Egrandis) |
| *Quercus robur* | <https://www.ncbi.nlm.nih.gov/labs/data-hub/taxonomy/38942/> |
| *Arabidopsis thaliana* | [https://www.arabidopsis.org/download/index-auto.jsp?dir=%2Fdownload_files%2FGenes%2FTAIR10_genome_release%2FTAIR10_chromosome_file](https://www.arabidopsis.org/download/index-auto.jsp?dir=/download_files/Genes/TAIR10_genome_release/TAIR10_chromosome_file) |
| *Oryza sativa* | <http://plants.ensembl.org/Oryza_indica/Info/Index> |
