# Supplementary material for: Populus cathayana genome and population resequencing provide insights into its evolution and adaptation
Source: Hortic Res. 2023 Dec 11;11(1):uhad255. doi: 10.1093/hr/uhad255 (PMC10809908; doi:10.1093/hr/uhad255)
Supplement: Web_Material_uhad255 [file web_material_uhad255.zip › 4. Supplementary methods_231031.docx]

Supplementary methods

Genome survey

Genomic DNA was extracted using DNeasy Blood &Tissue Kit (Qiagen). Pair-end libraries including 270 bp insert libraries were constructed by using Illumina’s paired-end kits. The libraries were sequenced on Illumina HiSeq X-10 platforms. For the raw reads, sequencing adaptors were removed; contaminated reads (chloroplast, mitochondrial, bacterial and viral sequences, etc.) were screened by alignment to NCBI-NR database using BWA v0.7.131 with default parameters; the FastUniq v1.12 was used to remove the duplicated read pairs [1]. Finally, we generated a total of 47.35 Gb clean reads for paired-end libraries, respectively. Corrected Illumina reads were selected to perform genome size estimation. Genome size estimation was conducted via a 19-kmer frequency analysis with JELLYFISH v2.1.4 [2]. The distribution of 19-kmer showed a major peak at 94× (Figure S1). The P. cathayana genome size was estimated using the formula: Genome size= kmer_Number / Peak_Depth. The above library construction and sequencing were platformed at BioMarker Technologies Co., Ltd. (Beijing, China).

RNA-sequencing

Total RNA of the leaves, shoots and roots from 1-month-old tissue culture plants was extracted with TRI Reagent (Sigma, St. Louis, MO) for transcriptome sequencing (RNA-seq). RNA-seq was performed on Illumina HiSeq X-10 platform. To assist gene annotation, we harvested leaves, stems, and roots from tissue culture. One sequencing library from mixed samples of these tissues was constructed using an Illumina standard mRNA-Seq Prep Kit.

Genome annotation

Protein-coding genes were predicted based on de novo-, protein homology- and RNA-Seq approaches. The de novo gene models were predicted using two ab initio gene-prediction software tools, Augustus (v 2.4) and SNAP (2006-07-28) [3]. For homology-based gene prediction, published protein sequences of six plant species, including *Populus trichocarpa* (v4, https://data.jgi.doe.gov/refine-download/phytozome?organism=Ptrichocarpa&expanded=Phytozome-533), *Populus euphratica* (v1,ftp://ftp.ncbi.nlm.nih.gov/genomes/all/GCF/000/495/115/GCF_000495115.1_PopEup_1.0), *Populus deltoides* WV94 (v2.1,https://phytozome-next.jgi.doe.gov/info/PdeltoidesWV94_v2_1), and *Populus alba* (v1, https://www.ncbi.nlm.nih.gov/data-hub/taxonomy/43335/), were downloaded and aligned onto the repat-masked genome by using the GeMoMa (v1.7) software [4]. For the transcript-based prediction, RNA-sequencing data were mapped to the reference genome using Hisat (v2.0.4) and assembled by Stringtie (v1.2.3) [5, 6]. GeneMarkS-T (v5.1) were used to predict genes based on the assembled transcripts [7]. The PASA (v2.0.2) software was used to predict genes based on the unigenes assembled by Trinity (v2.11). Gene models from these different approaches were combined using the EVM software (v1.1.1) (<http://evidencemodeler.sourceforge.net>) and updated by PASA [8]. The final gene models were annotated by searching the GenBank Non-Redundant (NR) (ftp://ftp.ncbi.nlm.nih.gov/blast/db), TrEMBL (http://eggnog5.embl.de/download/eggnog_5.0/), Pfam (http://pfam.xfam.org), SwissProt (http://ftp.ebi.ac.uk/pub/databases/swissprot), eukaryotic orthologous groups (KOG), gene ontology (GO) and Kyoto Encyclopedia of Genes and Genomes (KEGG) databases [9, 10] (Boeckmann et al., 2003; Kanehisa et al., 2016). Completeness of the genome was also assessed by performing core gene annotation using the Benchmarking Universal Single-Copy Orthologs (BUSCO) methods and Core Eukaryotic Genes Mapping Approach (CEGMA) methods [11].

TE annotation

Transposon element (TE) and tandem repeat were annotated by the following workflows. TE were identified by a combination of homology-based and de novo approaches. First, we customized a de novo repeat library of the genome using RepeatModeler, which can automatically execute two de novo repeat finding programs, including RECON (v1.08) and RepeatScout [12, 13]. Then full-length long terminal repeat retrotransposons (fl-LTR-RTs) were identified using both LTRharvest (-minlenltr 100 -maxlenltr 40000 -mintsd 4 -maxtsd 6 -motif TGCA -motifmis 1 -similar 85 -vic 10 -seed 20 -seqids yes) and LTR_finder (-D 40000 -d 100 -L 9000 -l 50 -p 20 -C -M 0.9). The high-quality intact fl-LTR-RTs and non-redundant LTR library were then produced by LTR_retriever [14]. Non-redundant species-specific TE library was constructed by combining the denovo TE sequences library above with the known Repbase (v19.06), REXdb (v3.0) and Dfam (v3.2) database. Final TE sequences in the P. cathayana genome were identified and classified by homology search against the library using RepeatMasker (v4.10). Tandem repeats were annotated by Tandem Repeats Finder and MIcroSAtellite identification tool (v2.1).

References

1. Xu H, Luo X, Qian J et al. FastUniq: a fast de novo duplicates removal tool for paired short reads. *PLoS One.* 2012; **7**:e52249. https://doi.org/10.1371/journal.pone.0052249

2. Marcais G, Kingsford C. A fast, lock-free approach for efficient parallel counting of occurrences of k-mers. *Bioinformatics.* 2011; **27**:764-770. https://doi.org/10.1093/bioinformatics/btr011

3. Stanke M, Diekhans M, Baertsch R et al. Using native and syntenically mapped cDNA alignments to improve de novo gene finding. *Bioinformatics.* 2008; **24**:637-644. https://doi.org/10.1093/bioinformatics/btn013

4. Keilwagen J, Hartung F, Grau J. GeMoma: homology-based gene prediction utilizing intron position conservation and RNA-seq data. *Methods Mol Biol.* 2019; **1962**:161-177. https://doi.org/10.1007/978-1-4939-9173-0_9

5. Kim D, Langmead B, Salzberg. HISAT: a fast spliced aligner with low memory requirements. *Nat Methods.* 2015; **12**:357-360. https://doi.org/10.1038/nmeth.3317

6. Pertea M, Pertea GM, Antonescu CM et al. StringTie enables improved reconstruction of a transcriptome from RNA-seq reads. *Nat Biotechnol.* 2015; **33**:290-295. https://doi.org/10.1038/nbt.3122

7. Tang S, Lomsadze A, Borodovsky M. Identification of protein coding regions in RNA transcripts. *Nucleic Acids Res.* 2015; **43**:e78. https://doi.org/10.1093/nar/gkv227

8. Haas BJ, Salzberg SL, Zhu W et al. Automated eukaryotic gene structure annotation using EVidenceModeler and the program to assemble spliced alignments. *Genome Biol.* 2008; **9**:R7. https://doi.org/10.1186/gb-2008-9-1-r7

9. Boeckmann B, Bairoch A, Apweiler R et al. The SWISS-PROT protein knowledgebase and its supplement TrEMBL in 2003. *Nucleic Acids Res.* 2003; **31**:365-370. https://doi.org/10.1093/nar/gkg095

10. Kanehisa M, Sato Y, Kawashima M et al. KEGG as a reference resource for gene and protein annotation. *Nucleic Acids Res.* 2016; **44**:D457-462. https://doi.org/10.1093/nar/gkv1070

11. Seppey M, Manni M, Zdobnov EM. BUSCO: assessing genome assembly and annotation completeness. *Methods Mol Biol.* 2019; **1962**:227-245. https://doi.org/10.1007/978-1-4939-9173-0_14

12. Bao Z, Eddy SR. Automated *de novo* identification of repeat sequence families in sequenced genomes. *Genome Res.* 2002; **12**:1269-1276. https://doi.org/10.1101/gr.88502

13. Price AL, Jones NC, Pevzner PA. *De* *novo* identification of repeat families in large genomes. *Bioinformatics.* 2005; **21 Suppl 1**:i351-358. https://doi.org/10.1093/bioinformatics/bti1018

14. Ou S, Jiang N. LTR_retriever: a highly accurate and sensitive program for identification of long terminal repeat retrotransposons. *Plant Physiol.* 2018; **176**:1410-1422. https://doi.org/10.1104/pp.17.01310
